# Mechanisms of Non-Vesicular Exchange of Lipids at Membrane Contact Sites: Of Shuttles, Tunnels and, Funnels

**DOI:** 10.3389/fcell.2021.784367

**Published:** 2021-11-29

**Authors:** Pascal F. Egea

**Affiliations:** Department of Biological Chemistry, David Geffen School of Medicine, UCLA, Los Angeles, CA, United States

**Keywords:** organelle, membrane contact site, lipid transfer protein, lipid distribution, membrane asymmetry, mitochondria-attached membranes, lipid-droplet, autophagy

## Abstract

Eukaryotic cells are characterized by their exquisite compartmentalization resulting from a cornucopia of membrane-bound organelles. Each of these compartments hosts a flurry of biochemical reactions and supports biological functions such as genome storage, membrane protein and lipid biosynthesis/degradation and ATP synthesis, all essential to cellular life. Acting as hubs for the transfer of matter and signals between organelles and throughout the cell, membrane contacts sites (MCSs), sites of close apposition between membranes from different organelles, are essential to cellular homeostasis. One of the now well-acknowledged function of MCSs involves the non-vesicular trafficking of lipids; its characterization answered one long-standing question of eukaryotic cell biology revealing how some organelles receive and distribute their membrane lipids in absence of vesicular trafficking. The endoplasmic reticulum (ER) in synergy with the mitochondria, stands as the nexus for the biosynthesis and distribution of phospholipids (PLs) throughout the cell by contacting nearly all other organelle types. MCSs create and maintain lipid fluxes and gradients essential to the functional asymmetry and polarity of biological membranes throughout the cell. Membrane apposition is mediated by proteinaceous tethers some of which function as lipid transfer proteins (LTPs). We summarize here the current state of mechanistic knowledge of some of the major classes of LTPs and tethers based on the available atomic to near-atomic resolution structures of several “model” MCSs from yeast but also in Metazoans; we describe different models of lipid transfer at MCSs and analyze the determinants of their specificity and directionality. Each of these systems illustrate fundamental principles and mechanisms for the non-vesicular exchange of lipids between eukaryotic membrane-bound organelles essential to a wide range of cellular processes such as at PL biosynthesis and distribution, lipid storage, autophagy and organelle biogenesis.

## 1 Introduction

Cellular compartments are present in both prokaryotic and eukaryotic cells; their compositions though are fundamentally different. While bacterial “organelles” are proteinaceous microcompartments essentially sequestering diverse biochemical pathways (e.g., CO_2_ fixation in the carboxysome of cyanobacteria) (Yeates et al., 2008; Chowdhury et al., 2014; Kerfeld and Erbilgin, 2015; Nichols et al., 2017), most eukaryotic organelles are delineated by one (e.g., ER and Golgi), two (e.g., nucleus, mitochondria, chloroplasts) or sometimes four membranes (e.g., apicoplasts in the Plasmodium and Toxoplasma parasites) and simultaneously carry out numerous functions (e.g., oxidative phosphorylation, lipid biosynthesis, photosynthesis). More recently a new class of organelle, found in Prokaryotes and Eukaryotes, has been defined as “membrane-less organelle” based on their liquid-liquid phase separation (LLPS) and reversible gel-like behavior. They have the ability to selectively sequester macromolecules such as proteins/enzymes and nucleic acids (RNAs) and the biological pathway associated with these trapped enzymes and ribonucleoproteins (RNPs); the presence of intrinsically disordered protein regions and low-complexity regions (LCRs) has been shown to be essential to these processes (Han et al., 2012; Kato et al., 2012; Wright and Dyson, 2015; Gomes and Shorter, 2019). The CO_2_-fixing pyrenoid found in the chloroplast stroma of algae is one last remarkable example of “organelle” with a mysterious internal tubular membrane backbone although being essentially based on a surrounding LLPS that concentrates CO_2_ to saturate its sequestered Rubisco, the main enzyme responsible for CO_2_ fixation and conversion into biomass (Wunder et al., 2019; Barrett et al., 2021). While compartmentalization is not unique to eukaryotic cells, its level of sophistication, complexity, plasticity and functional integration seemingly surpasses what prokaryotic cells have achieved since the emergence and evolution of life on Earth. Compartmentalization supports highly specialized functions but also creates new challenges such as the need for specific and efficient mechanisms for the transfer of matter and information between compartments and throughout the cell in response to diverse intracellular and extracellular cues; this integration of a “global organelle network” is essential to cellular and organismal life.

In 2013, the Nobel prize in Physiology or Medicine was awarded for “the discoveries of machineries regulating vesicle traffic, a major transport system in our cells.” The Rothman, Südhof and Schmekan groups characterized the molecular machineries and mechanisms for the energy-dependent vectorial transport of macromolecules between membrane-enclosed organelles through their encapsulation into small membranous vesicles. Their work proved the hypothesis originally posited by the 1974 Nobel laureate George Emil Palade (Bonifacino, 2014). While vesicular transport mediates delivery of diverse cargo such as proteins but also lipids in the endomembrane secretory system, the mechanisms explaining lipid fluxes between most organelles from yeasts to humans have long remained elusive.

**FIGURE 1:**
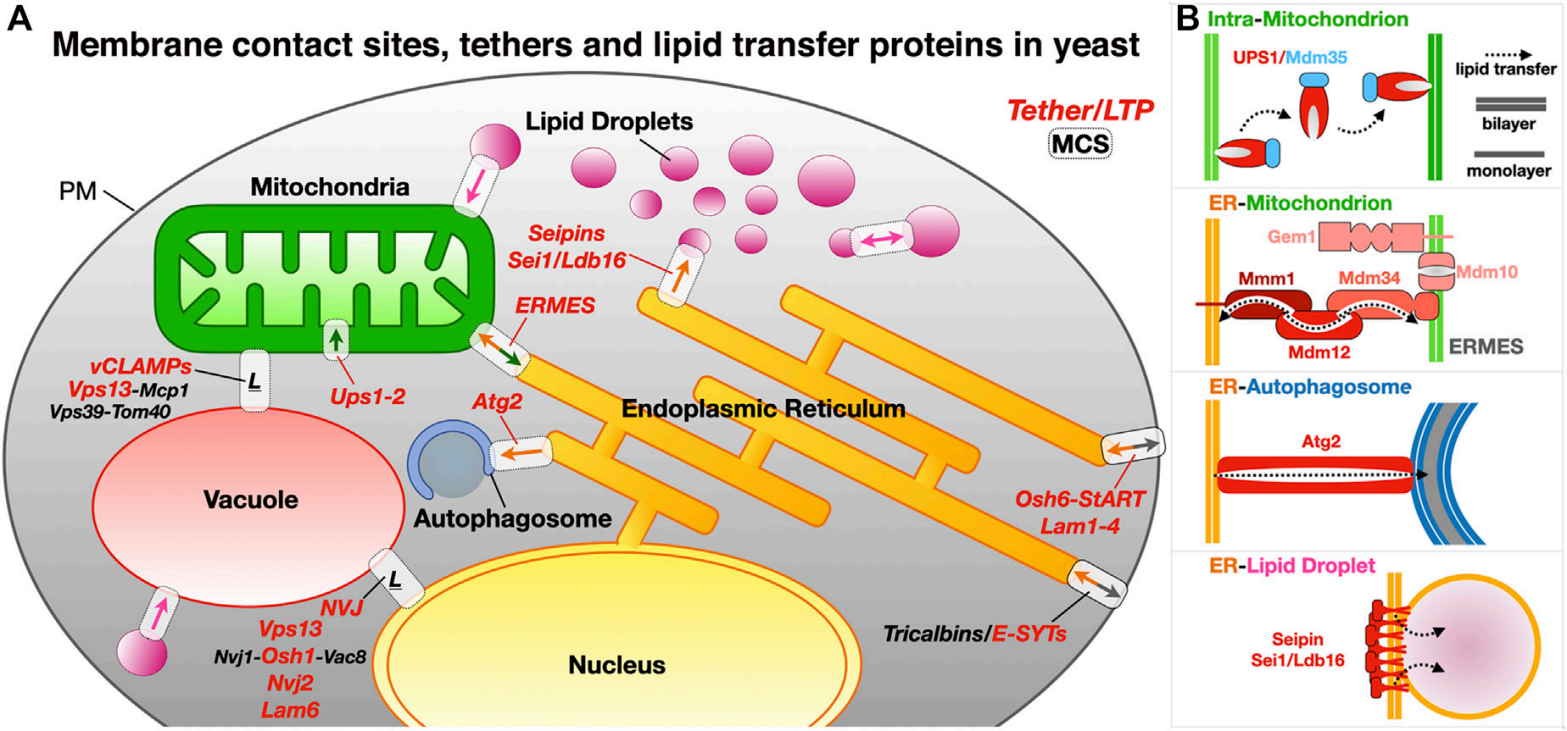
Membrane contact sites and their associated proteinaceous tethers in yeast. **(A)** Schematic of a yeast cell showing some of the MCSs existing between diverse organelles together with some of the identified proteinaceous tethers mediating juxtaposition of their membranes. ER, endoplasmic reticulum; PM, plasma membrane; LD, lipid droplets; ERMES, ER-mitochondrion encounter structure; vCLAMP, vacuole and mitochondria patch; NVJ, nuclear ER-vacuole junctions. Ups/Mdm35, at the inner mitochondrial membrane space interface (IMMSI); Atg2 at the ER-to-autophagosome contact site; Vps13 at vCLAMP and NVJ. Lam6, NVJ protein 2 and NVJ protein 1 associated to Vac8 and Osh1 at NVJ; Seipins at ER-LD contact sites (yeast and Metazoans); Tricalbins/E-SYTs (yeast/Metazoans) at ER-PM contact sites. Colored arrows indicate directionality of lipid exchange. **(B)** Schematic organization of some of the tethers/LTPs at the MCSs described as examples.

Although observed since the 1950s, membrane contact site, sites of tight apposition between membranes from different organelles (heterotypic apposition), have emerged as major platforms for the exchange of lipids and other molecules and signals (such as calcium ions) within the eukaryotic cell but also the regulation of many other complex biological pathways (Achleitner et al., 1999). This transfer “*sans*” vesicles is assisted or catalyzed by proteins and protein complexes that function as exchange factors between heterotypic membranes. MCSs in yeast and human cells have been extensively imaged *in vivo* to characterize their morphology, size, cellular repartition and dynamics (Valm et al., 2017; Guo et al., 2018; Scorrano et al., 2019; Hoffman et al., 2020); they can be distinguished as static or dynamic but also as functional, regulatory or architectural (i.e., participating to the cellular ultrastructure) (Scorrano et al., 2019). The average distance between apposed membranes ranges between ∼150 and 250 Å. The protein tethers responsible for the juxtaposition of membranes between different organelle types and their mechanisms of action have been dissected using biochemical, genetic and structural approaches combining X-ray crystallography and cryo-Electron Microscopy (cryo-EM) or cryo-Electron Tomography (cryo-ET) both *in vitro* and *in vivo* (Csordás et al., 2006; Csordás et al., 2010; Friedman and Voeltz, 2011; Friedman et al., 2013; Fernández-Busnadiego et al., 2015; Phillips and Voeltz, 2016; Kwak et al., 2020) in yeast but also Metazoans (Figure 1).

In the eukaryotic cell, making and distributing PLs requires sharing. As a central player in the biosynthesis of lipids and membrane proteins, the ER contacts virtually all other organelles from mitochondria, Golgi to lipid droplets (LDs), vacuole/lysosome and endosomes. Lipids and in particular PLs are important to many aspects of cellular life. They are the main components of biological membranes defining the very boundaries of cells and their organelles where their physicochemical properties determine the fluidity, plasticity/rigidity, permeability and shape/curvature of these membranes. They also serve as energy stores (e.g., triacylglycerides and sterol-esters in LDs) or signaling molecules (e.g., phosphoinositides, diacyl-glycerides, sterols). While some of them are consumed for the purpose of fueling and maintaining cellular energetics, lipids also play essential regulatory roles by affecting the structure and function of membrane proteins (Li et al., 2021); for example cardiolipins are lipids primarily found in the inner mitochondrial membrane and are essential to the function of the respiratory chain enzymes (Dudek et al., 2019).

**FIGURE 2:**
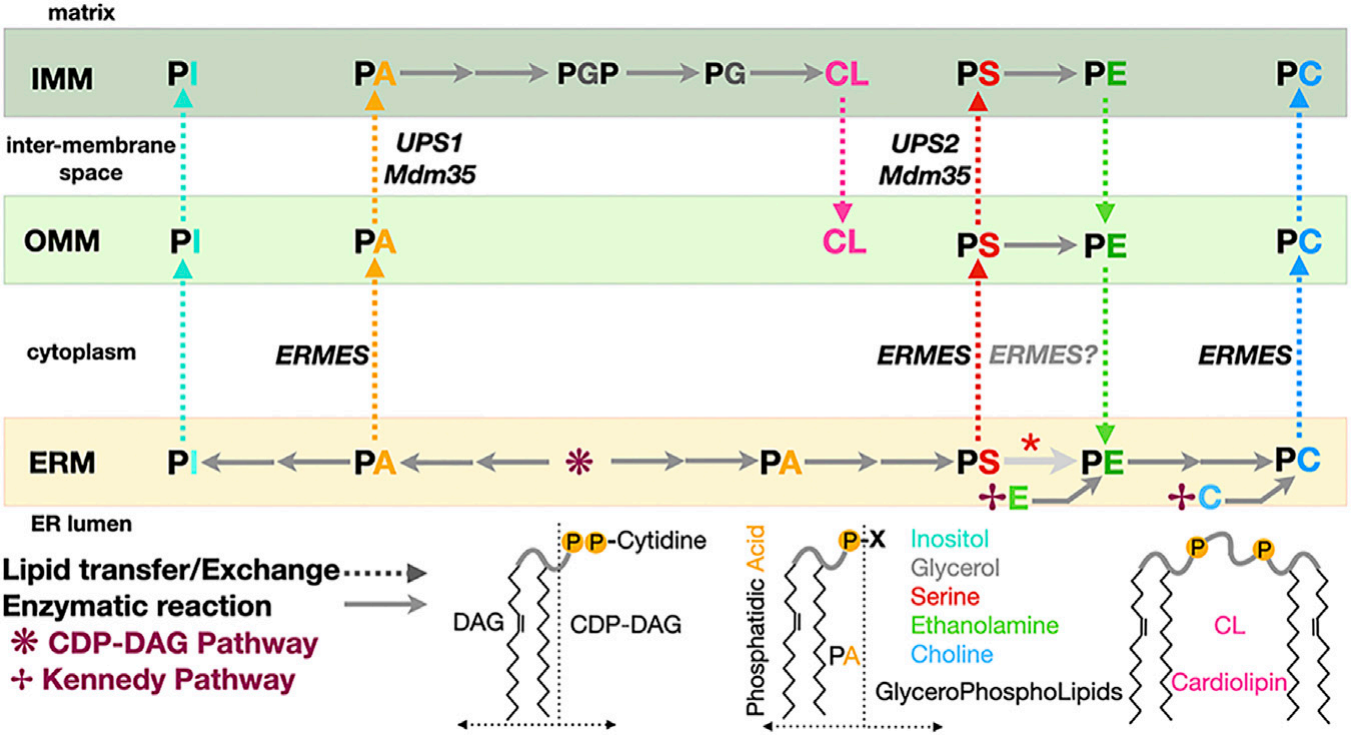
Phospholipid biosynthesis is shared between the endoplasmic reticulum and the mitochondrion. Simplified pathway for the biosynthesis of the major classes of glycerophospholipids in yeast. PA, phosphatidic acid; PE, phosphatidyl ethanolamine; PS, phosphatidyl serine; PC, phosphatidyl choline; PG, phosphatidyl glycerol; PGP, phosphatidyl glycerol phosphate; PI, phosphatidyl inositol; CL, cardiolipin. CDP-DAG, CDP diacylglycerol. DAG and its “activated” form CDP-DAG are some of the main building blocks providing the lipidic backbone of the PLs subsequently synthetized. Two pathways contribute of PL biosynthesis in yeast: the CDP-DAG pathway to generate PA and then PS subsequently transformed into PE then PC and the Kennedy pathway using DAG and ethanolamine or choline to directly generate PE or PC. Dotted arrows indicate PL transfer between membranes within the mitochondrion or between ER and mitochondrion; continuous arrows indicate biosynthetic reactions. A red asterisk indicates that depending on the carbon source, decarboxylation of PS into PE can be observed in the ER membrane. Some of the lipid exchange proteins or membrane tethering complexes (i.e., Ups1/Mdm35 and ERMES) shown to be involved in specific transfer/exchange are indicated.

Organelle membranes have very distinct lipid compositions (van Meer et al., 2008) as a result of the compartmentalization of lipid biosynthetic pathways and of vesicular and non-vesicular trafficking. Furthermore, membranes are asymmetrical and heterogenous: Integral membrane proteins known as lipid scramblases and lipid flippases/floppases affect the repartition of lipids (in particular PS) between leaflets and the creation of lipidic micro or nanodomains (Montigny et al., 2016; Shin and Takatsu, 2020; Lenoir et al., 2021). The creation and maintenance of lipid fluxes and gradients and the resulting membrane asymmetry are essential to the establishment of organelle identity and a functional organelle network. The ER is the main site of PL biosynthesis and thus central supplier to the rest of the cell, while mitochondria use a considerable amount of PLs for their membrane biogenesis; the PL biosynthetic pathway in eukaryotes in general and in yeast in particular share a remarkable feature (Horvath and Daum, 2013; Tamura et al., 2014; Tatsuta et al., 2014; Tamura and Endo, 2017; Acoba et al., 2020) where the ER and mitochondria depend on each other for the exchange of intermediates necessary to its completion (Figure 2). While some lipids can be consumed to produce energy, cells have also evolved autophagic pathways involving membrane-based structures (i.e., phagosomes) to recycle and salvage the membrane components and the contents from damaged or decaying organelles. Here we survey some of the general mechanistic aspects of lipid transfer and exchange at MCSs in yeast but also Metazoans and even bacteria at the level of the molecular structure of the diverse protein and protein complex effectors elucidated at atomic or near-atomic resolution. The structures described herein have all been deposited at the *Protein Data Bank* (www.rcsb.org) and the *Unified Data Resource for 3D-EM* (www.emdataresource.org) and are referenced with their identifiers throughout this review.

## 2 Lipid Transfer Proteins: A Large Functional Group of Proteins Sampling Many Structural Classes

### 2.1 A “Menagerie” of Lipid Transfer Proteins

**FIGURE 3:**
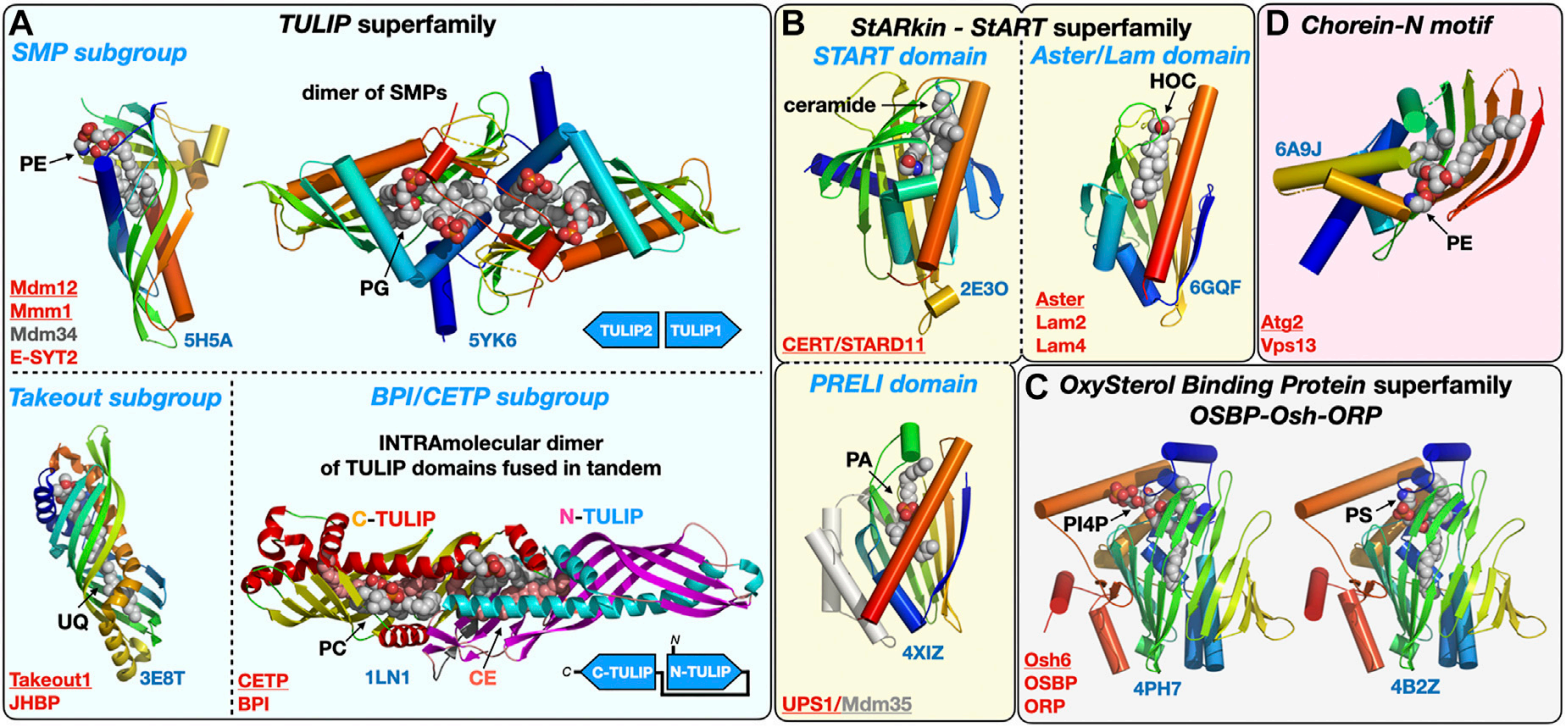
Structural gallery of protein folds and domains observed in lipid-transfer proteins. **(A)** TULIP protein superfamily representatives within the three subgroups: The SMP domain found in ERMES subunits Mdm12, Mmm1 and Mdm34 and in Extended Synaptotagmins (E-SYT)/Tricalbins (Tcb); the structures of the SMP domains from Mdm12 and Mmm1 (dimer) are shown. The Takeout and BPI/CETP subgroups. **(B)** StARkin protein superfamily with StART and StART-like domains represented by the ceramide transporter CERT/STARD11 and the sterol-binding Aster/Lam proteins, respectively, and also including the PRELI domain represented by the Ups1/Mdm35 PA shuttle in the mitochondrial intermembrane space. **(C)** OxySterol Binding Protein superfamily including Osh and ORP proteins, Osh6 is represented bound to the two lipids (PS and PI4P) it counter-exchanges at ER-PM junctions. **(D)**. Chorein-N motif found in proteins Vps13 and Atg2 involved in bulk lipid transfer at several MCSs and the autophagosome, respectively. The motif present in the Atg2 N-terminal fragment crystal structure was observed bound to a PE molecule. Ligands are represented with spheres. Proteins are colored with a rainbow pattern except for the CETP/BPI protein where the two TULIP domains present in tandem are colored differently to highlight the head-to-head intramolecular arrangement of the two TULIP domains. UQ, ubiquinone; CE, cholesteryl-ester; PI4P, phosphatidyl-inositol 4-phosphate; HOC, hydroxycholesterol. Underlined names correspond to the displayed structures (PDB 5H5A-5YK6-3E8T-1LN1-4XIZ-2E3O-6GQF-4PH7-4B2Z-6A9J).

Many lipid transfer proteins are found at MCSs where they facilitate the exchange of lipids between organelles. They can function as tethers or be co-opted by tethers or adaptor proteins that recruit them at specific sites thus imparting regiospecificity to the lipid transfer activity they carry out. However, not all tethers are LTPs and not all LTPs are tethers. LTPs constitute a large and structurally diverse functional class of proteins (Wong et al., 2017; Wong et al., 2019), encompassing members from several protein (super)families with their associated lipid-binding protein fold such as the Steroidogenic Acute Regulatory-lipid related Transfer (StART) proteins and their StART-like relatives (StARkin) (Alpy and Tomasetto, 2014; Wong and Levine, 2016; Horenkamp et al., 2018; Jentsch et al., 2018) or the Tubular Lipid-binding domain superfamily (TULIP) (Alva and Lupas, 2016; Wong and Levine, 2017) that includes the subgroup of Synaptotagmin-like Mitochondrial lipid-binding Protein domain (SMP) (Lee and Hong, 2006) exclusively associated with MCSs (Toulmay and Prinz, 2012) (Figure 3). The TULIP superfamily also includes the *Takeout* and *BPI/CETP* subgroups. Takeout and similar proteins such as the Juvenile Hormone Binding Protein (JHBP) serve as hydrophobic ligand carriers in the hemolymph of insects (Hamiaux et al., 2009; Suzuki et al., 2011) while the Cholesteryl Ester Transfer Protein (CETP) transports cholesteryl esters, triglycerides and PLs in blood plasma (Qiu et al., 2007); these two subgroups thus encompass hydrophobic ligand carrier extracellular proteins but make use of the same protein fold.

Yeast Oxysterol homology (Osh) proteins, mammalian OxySterol Binding Protein (OSBP) and Oxysterol-binding Related Proteins (ORP) proteins form another large LTP protein family (Kentala et al., 2016). At least some 22 crystal structures of LTP domains from Osh or ORP proteins have been tallied so far including Osh1 (Manik et al., 2017), Osh3 (Tong et al., 2013), Osh4 (Im et al., 2005; de Saint-Jean et al., 2011), Osh6 (Maeda et al., 2013; Moser von Filseck et al., 2015), ORP1, ORP2 (Wang et al., 2019) and ORP3 (Tong et al., 2021) bound to different phosphoinositides and/or cholesterol derivatives; these structures reveal the versatility of this particular LTP fold capable of accommodating different types of ligands (e.g., Osh6 binding PS or PI4P and OSBP binding cholesterol or PI4P). A similar versatility can be described for another large class of LTPs including the PRELI and StART/StARkin domains (Alpy and Tomasetto, 2014) (Figure 3) capable of binding a variety of lipids such as PLs (PA or PS) and sterols or ceramides, respectively.

### 2.2 Three Basic Mechanisms of Lipid Transfer: Shuttling, Sliding and Bridging/Tunneling

**FIGURE 4:**
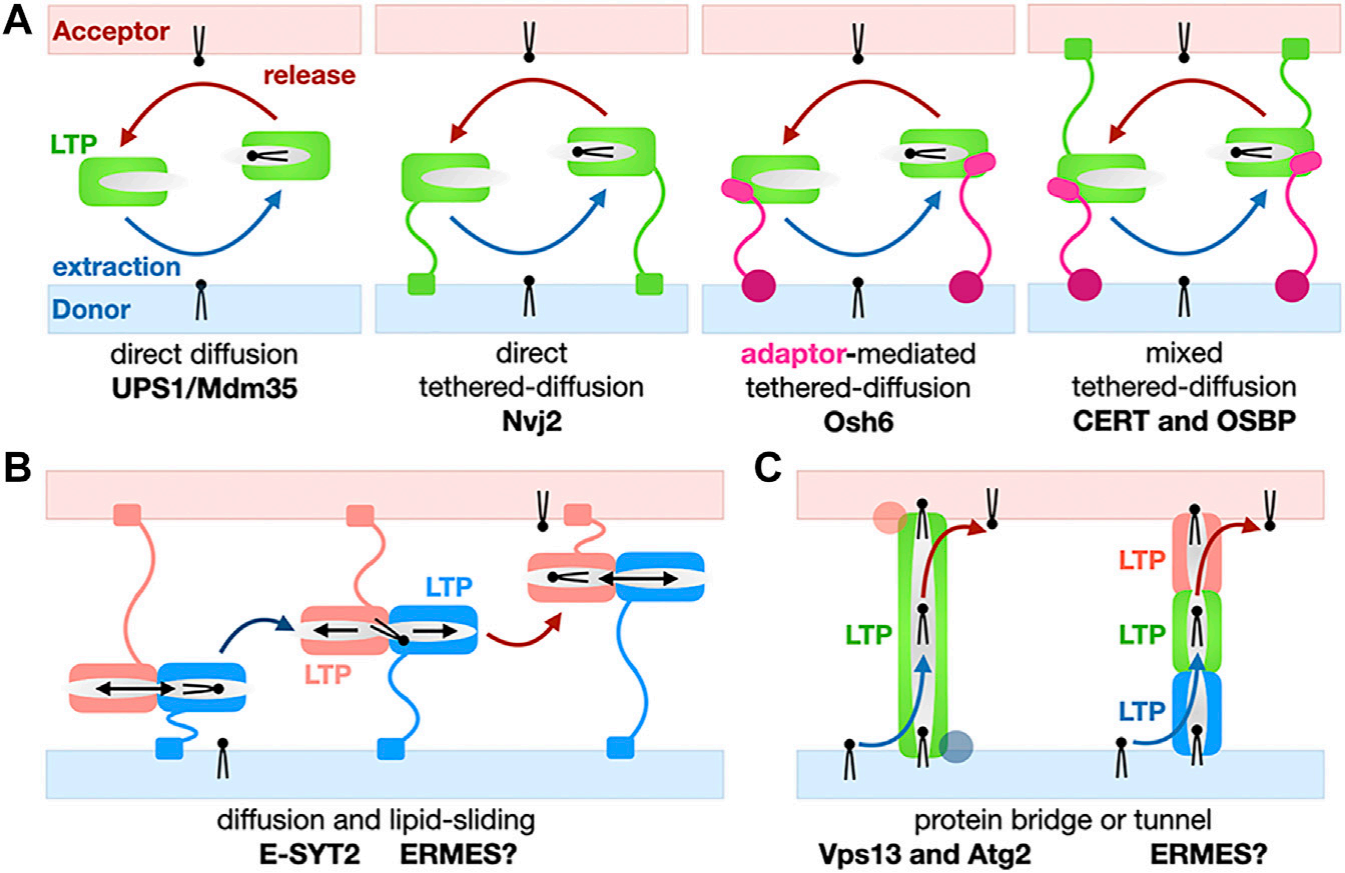
Basic mechanisms for the non-vesicular exchange of lipids using lipid transfer proteins. **(A)** Transfer based on protein domain diffusion. Variations on the use of a soluble LTP domain for the shuttling of lipids between membranes: Soluble *versus* tethered LTP shuttles with or without combination of protein motifs and/or adaptor proteins targeting the LTP module to a specific MCS. Membrane-tethering motifs on the LTP or the adaptor can be present on both membranes. Only monovalent tethers/adaptors are shown here for the sake of simplification but more complex examples have been described. **(B)** Transfer using lipid sliding within a LTP domain combined with diffusion. Here we show a general simplified model; E-SYT2 (and possibly ERMES?) fall under this general category. **(C)** Transfer based on lipid bridges/tunnels. The lipid conduit spans the entire space separating the two organelles at a MCS can consist of one large LTP/tether protein (Vps13 and Atg2) or several LTPs (possibly SMP domains in ERMES?).

Although not all LTPs are present at MCSs they all function by extracting/releasing lipids from and into membranes and shielding them from the aqueous phase by burying them in their protein core during the transfer step between the donor and acceptor organelles. The structural diversity of LTPs also translate into a certain diversity of their mechanisms of action. Mechanisms of non-vesicular transfer of lipids by LTPs roughly fall into three classes simplistically described in Figure 4. Each mechanism is exemplified and analyzed in more depth in the following Sections 3 through 6 of this review; the boundaries between categories are however not set in stone as some lipid exchange systems combine several of these mechanisms. LTPs can consist of a “simple” lipid transfer domain or have a more complex multi-domain architecture including several lipid exchange domains and/or other protein modules involved in specific membrane-binding and/or interaction with other membrane associated or embedded proteins thus determining their cellular location and tethering capabilities. Despite the structural diversity of LTPs, a few common features emerge: Their lipid transfer domains are usually ∼150–300 amino acids in size and use highly twisted β-sheets to fold into basket, barrel or cup -shaped structures with binding pockets lined with hydrophobic residues to accommodate the aliphatic hydrocarbon chains of lipids. Some of these structures resemble baskets, sealed at one end and with flexible loops or small helices acting as lids or gates at their entrance to reversibly entrap and release lipidic ligands and function as shuttles (Figure 4A) (e.g., Ups1/Mdm35 or Osh/ORP, StART/StARkin).

Others LTPs are tubular-shaped (e.g., SMP domains of the TULIP family) with a hydrophobic tunnel seemingly suited for the channeling of lipids from one end to the other by lipid sliding (Figure 4B). PLs bear charges as they all harbor negatively charged phosphate moieties but also other charged head groups such as serine, ethanolamine (decarboxylated serine) and choline (trimethylated ethanolamine). This energetic consideration is also relevant to the extraction/desorption steps where a lipid passes from a membrane into a proteinaceous binding pocket and vice versa). Because of the energetic penalty associated with burying polar headgroups into a hydrophobic cavity, some of these tubular LTPs display a lateral opening (also referred to as “seam”) that runs along the tube/barrel and whose edges are sometimes delineated by twisted α-helices. This solvent-accessible seam, accommodates the hydrophilic headgroups while aliphatic chains remained buried within the LTP core and enables the sliding of lipids alongside the tubular core of the LTP (Figure 3).

Lipid-conducting *protein bridges* or *tunnels* such as Vps13 and Atg2 that will be discussed in Section 5 constitute one spectacular exception as these LTPs*-*tethers are much larger (∼1,500–3,000 residues for yeast Atg2 and Vps13, respectively) and elongated (∼200–400Å) proteins: Their lipid transfer domains extend beyond their small lipid-binding signature Chorein-N motif (Figures 3, 4C).

## 3 Lipid Transfer by a Diffusive LTP Shuttle at the Mitochondrial Inter-membrane Space

**FIGURE 5:**
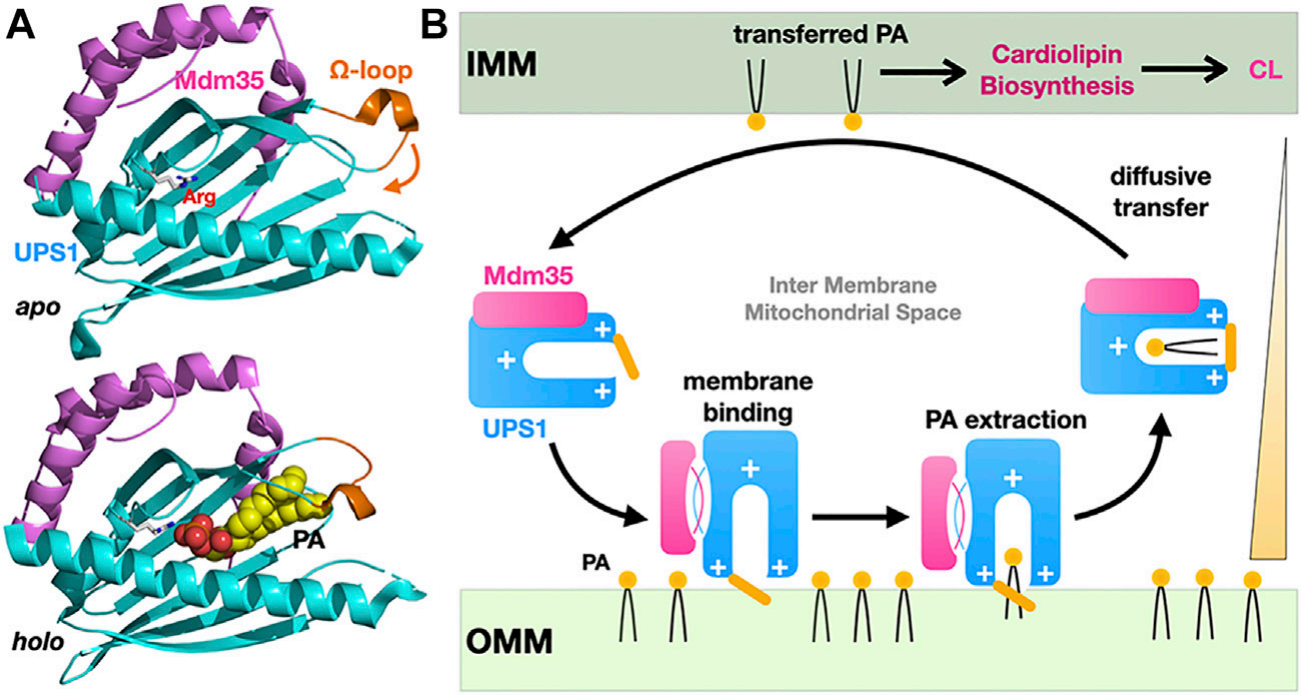
Diffusive transfer of phosphatidic acid between mitochondrial membranes by the soluble LTP Ups1/Mdm35. **(A)** Structures of the *apo* and PA-bound Ups1/Mdm35 soluble LTP complexes emphasizing the polar arginine clasp (sphere) at the bottom of the PA-binding pocket, the Ω-loop (orange) sealing the hydrophobic cavity entrance and involved in membrane attachment and PA extraction. The *apo* and *holo* structures are shown in the same orientation. **(B)** Simplified cycle for the diffusive LTP-assisted transfer of PA between mitochondrial membranes. Mdm35 reversible dissociation from Ups1 enhances its membrane association. Diffusive transfer is driven by the PA concentration gradient as transferred PA fuels CL biosynthesis (PDB 5QJM-5YTX).

The example of the yeast Ups1-Mdm35 phosphatidic acid (PA) mitochondrial shuttle illustrates the basic principles for how a soluble LTP confined between two closely apposed membranes at a MCS can function as a “simple” shuttle without the need of being physically tethered to one or both membranes to promote efficient vectorial transfer of a PL along its concentration gradient. Ups1 is the LTP subunit while Mdm35 is a regulatory subunit controlling mitochondrial import of Ups1, its sensitivity to proteolytic degradation and its affinity towards membranes during the PA transfer cycle (Figure 5A). A second mitochondrial shuttle system, Ups2-Mdm35, transports PS instead of PA using the same diffusive mechanism (Miyata et al., 2016). Studies based on crystal structures of apo and lipid-bound Ups1/Mdm35 complexes, biochemical/mutational analysis and, molecular dynamic simulations (Watanabe et al., 2015; Yu et al., 2015; Lu et al., 2020) have shown the importance of an Ω-loop gating the entrance of the PA-binding pocket and of electrostatic interactions for membrane-binding and PA extraction/desorption from the membrane (Figure 5B). Similar studies on the PI4P or PS/sterol counter-exchange Osh4 (von Filseck et al., 2015) and Osh6 (Moser von Filseck et al., 2015) LTPs have shown that switching electrostatic interactions in the anionic lid gating its binding pocket regulate its interactions with membrane at ER-PM contact sites as capture of PS and P4IP results in lid closure and lowers its avidity towards and residency time on membranes (Lipp et al., 2019).

While the vectorial efficient transfer of PA by a soluble LTP such as Ups1/Mdm35 operating in the constrained and “closed” MIMS compartment is simple to conceptualize, other shuttle systems working in the context of more “open” MCSs in the cytosol function with equal efficiency but use tethering to localize and target the transfer process to a specific MCS. Several variations of the simple direct diffusion LTP model exist: The LTP domain is directly tethered to one or both membranes at the MCS, one or several membrane-associated adaptor(s) can tether the LTP domain to the MCS and combinations of these two models have also been observed (Figures 4A,B); adaptors/membrane attachment domains often include (phospho)lipid-binding modules conferring high localization specificity (Lemmon, 2008). Variations on these common themes have been characterized at the cellular and structural levels in yeast or human cells; such LTP systems dissected in detail include, the SMP-containing E-SYT2 shuttle of glycerophosholipids at ER-PM MCSs (Schauder et al., 2014; Saheki et al., 2016), CERT/STARD11 transporting ceramides (Hanada et al., 2003; Kudo et al., 2008) between ER and Golgi and the Aster proteins (Sandhu et al., 2018) transferring cholesterol from PM to ER in cholesterol-depleted membranes both belonging to the StART/StARkin group of LTPs. Other seminal studies of the Osh6 (Maeda et al., 2013; Moser von Filseck et al., 2015) and OSBP (Mesmin et al., 2013; Antonny et al., 2018) ORP LTPs responsible for the PS- or cholesterol- PI4P counter-exchanges at ER-to-PM and ER-to-Golgi MCSs, respectively, will be discussed in Section 7 of this review to analyze the energetics and thermodynamics of lipid exchange at MCS.

## 4 The Perplexing Case of ERMES and its SMP Domains at Mitochondria-Associated Membranes

The role of mitochondria-associated membranes in lipid trafficking in yeast (and later in metazoans) has been described several decades ago (Simbeni et al., 1990; Simbeni et al., 1991; Simbeni et al., 1993; Gaigg et al., 1995). Since then, studies have revealed their involvement in many other essential eukaryotic cellular processes relevant to human health and diseases. In yeast, one of the better characterized effectors is the ER-to-Mitochondrion Encounter Structure (ERMES) that uses SMP domains to mediate lipid exchange.

### 4.1 The ER-to-Mitochondrial Encounter Structure, a Multi-Functional Tether

**FIGURE 6:**
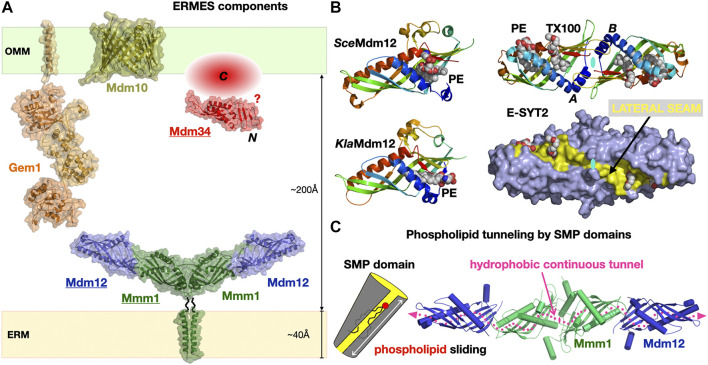
The Endoplasmic Reticulum-to-Mitochondrion Encounter Structure. **(A)** Composite structural model of the yeast ERMES including the structures of the Mdm12/Mmm1 heterotetrameric core of SMP domains and the Mmd10 porin and combining the homology models of the Gem1 Miro GTPase and the Mdm34 SMP domain generated in *Phyre* ^*2*^. The C-terminal domain of Mdm34 is represented as an unstructured module. **(B)** Structures of diverse SMP domains -Mdm12 (*S. cerevisiae* and *K. lactis*) and dimer of E-SYT2- showing the different positions sampled by bound PLs along the lateral seam of the SMP domain; the E-SYT2 dimer is represented using ribbon and also full surface (slate blue) representations to emphasize the existence of a lateral seam (yellow). PL ligands are shown as spheres; in E-SYT2 an additional molecule of Triton X100 detergent was observed. All structures are shown under the same orientation relative to E-SYT2 monomer A. **(C)** Hydrophobic tunnel spanning the entire length of the Mdm12/Mmm1 tetrameric assembly of aligned SMP domains for the sliding of PLs along the solvent-accessible lateral seam (magenta dotted line) exposing the polar headgroups of the PLs (PDB 5GYD-5H5A-4P42-5YK7).

ERMES was initially discovered by the Walter group using a synthetic genetic screen approach (Kornmann et al., 2009) and shown to be composed of the mitochondrial porin Mdm10 and of three SMP domain containing subunits: cytosolic Mdm12, ER-anchored Mmm1 and mitochondrial-attached Mdm34. Later on, a fifth subunit the calcium-binding GTPase Gem1, an ortholog of Miro GTPases was identified (Kornmann et al., 2011; Stroud et al., 2011) (Figure 6A). ERMES involvement in PL exchange (transfer of PS and PC) at the ER-to-Mitochondria interface has now been established combining *in vitro* (Kawano et al., 2017; Jeong et al., 2017) and *in vivo* approaches (Kojima et al., 2016). ERMES is not essential though as dominant mutations in Vps13 (Lang et al., 2015; John Peter et al., 2017), another LTP found at nucleus-to-vacuole junctions (NVJ) (Malia and Ungermann, 2016) and vacuole-and-mitochondria patch (vCLAMP) (Elbaz-Alon et al., 2014; Hönscher et al., 2014) MCSs can compensate for the lack of ERMES function. Furthermore, two different complexes have been identified at vCLAMPs one with Vps13 and one with Vps39 (González Montoro et al., 2018). Vps39 is also part of the dynamic HOPS (homotypic fusion and protein sorting) and CORVET tethering complexes (Balderhaar and Ungermann, 2013) involved in endosomal tethering (thus involving a vesicular trafficking pathway) while a dynamic exchange of/competition for Vps39 between HOPS and vCLAMP affects contact site formation at the vacuole (Montoro et al., 2021). These studies reveal the existence of redundant pathways (i.e., ERMES, Vps13-vCLAMP and Vps39-HOPS but not Vps39-vCLAMP) supporting lipid fluxes the cell. In yeasts deficient for the ER PS synthase, heterologous ectopic expression of bacterial PS synthase targeted to peroxisomes or LDs shows that PS reaches mitochondria to be transformed into PE (Shiino et al., 2020). Another recent study combining the simplification and systematic rewiring of PE/PC biosynthesis in yeast using enzyme retargeting (Figure 2) and saturated transposon mutagenesis analysis has elegantly revealed the robustness of lipid cellular distribution together with the existence of lipostatic adaption mechanisms (Peter et al., 2021) that remain to be studied. Other ERMES functions include involvement in mitochondrial protein import and assembly (Ellenrieder et al., 2017), calcium signaling and regulation of its cellular pools, distribution of mitochondria and their DNA (Friedman et al., 2011; Murley et al., 2013), iron homeostasis (Xue et al., 2017) and coenzyme Q biosynthesis (Eisenberg-Bord et al., 2019).

#### 4.1.1 Deceptively Tubular SMP Domains

SMP domains are exclusively associated with MCSs (Toulmay and Prinz, 2012; Jeyasimman and Saheki, 2020); however, ERMES is not found in Metazoans and seems mostly restricted to yeasts and fungi (Wideman et al., 2013). SMP domains were found in yeast tricalbins (Manford et al., 2012; Qian et al., 2021), the orthologs of Extended Synaptotagmin proteins (E-SYTs) in Vertebrates (Saheki and De Camilli, 2017) at ER-to-PM junctions (Fernández-Busnadiego et al., 2015) and also in PDZ domain-containing protein 8 (PDZD8), first at ER-to-mitochondria (Hirabayashi et al., 2017) then at ER-to-late endosome/lysosomes interfaces (Guillén-Samander et al., 2019; Elbaz-Alon et al., 2020; Shirane, 2020; Shirane et al., 2020) also in Vertebrates. PDZD8 thus appears to be a shared component at a three-way MCS between ER, mitochondria and, late endosomes. The structure of the E-SYT2 SMP domain (Schauder et al., 2014) was the first to reveal the fold of this member of the TULIP protein superfamily (Figure 3). The structures of the stable Mdm12-Mmm1 subcomplex elucidated by negative-stain EM (NS-EM) (AhYoung et al., 2015) and X-ray crystallography (Jeong et al., 2017) and several structures of Mdm12 (Kawano et al., 2017; Jeong et al., 2016; AhYoung et al., 2017) from different yeasts also shed light on the dynamics of the SMP-PL interaction. In particular, the positions sampled by the PLs observed in the SMP of Mdm12 strongly suggest a “sliding” mechanism where PLs can move along the lateral seam of the SMP domain with their polar head exposed to the solvent while the acyl chains remain buried in the central hydrophobic tunnel (Figure 6B). The Mdm12-Mmm1 tetramer is stable and relatively rigid although some alteration of the curvature of the crescent-shaped assembly is noticeable when comparing EM and X-ray structures. The domain arrangement quasi aligns the central tunnels of each SMP module resulting in a nearly continuous central hydrophobic channel. Mutations introduced at the Mmm1-to-Mdm12 interface (Jeong et al., 2017) blocked lipid exchange *in vitro* thus supporting this model of a lipid sliding mechanism through a long composite central channel. The curvatures observed in the E-SYT2 dimer and Mdm12-Mmm1 tetramer, might be important at the lipid desorption step to modulate membrane shape (and vice-versa). So far, the mechanistic importance and roles of the two-fold symmetry displayed within the Mdm12-Mmm1 tetramer remain unclear.

Within ERMES, the structures of Mdm12 and Mmm1 SMPs have informed us on their likely function and mechanism of action during PL exchange and transfer. The role of the more perplexing Mdm34 remains mysterious. Mdm34 contains an N-terminal SMP domain, shown to dimerize *in vitro* (AhYoung et al., 2015), followed by a region of variable length and with no identifiable structural homologue. Because Mdm34 localizes to the surface of mitochondria, it is assumed that its uncharacterized C-terminal region has some kind of affinity for membranes, specifically anchoring its SMP to the mitochondrion; one other possibility is that Mdm34 interacts with Mdm10 and this mediates its localization to the mitochondrial outer membrane surface. Sequence analysis indicate that the “tips” of the three SMP tubulars domains in ERMES correspond to conserved surfaces (AhYoung et al., 2015); this agrees with the observed mode of interactions for the “head-to-head” homodimeric association of Mmm1 and the “head-to-toe” Mdm12/Mmm1 heteromeric association (Jeong et al., 2017) (Figure 6). In the Mdm12/Mmm1 tetramer each “toe” of the Mdm12 subunits is available to establish potential interactions with Mdm34. Nevertheless, the role of the C-terminal “domain” of yeast Mdm34 is unclear. Structure prediction computational tools suggest that this region is disordered; although this is not an LCR, it is unclear if it is intrinsically disordered and could promote LLPS or if this represents another case where structure prediction computational tools fail to detect any clear homology with other known proteins in structural databases.

#### 4.1.2 Models for SMP-Facilitated Lipid Transfer Between Membranes

**FIGURE 7:**
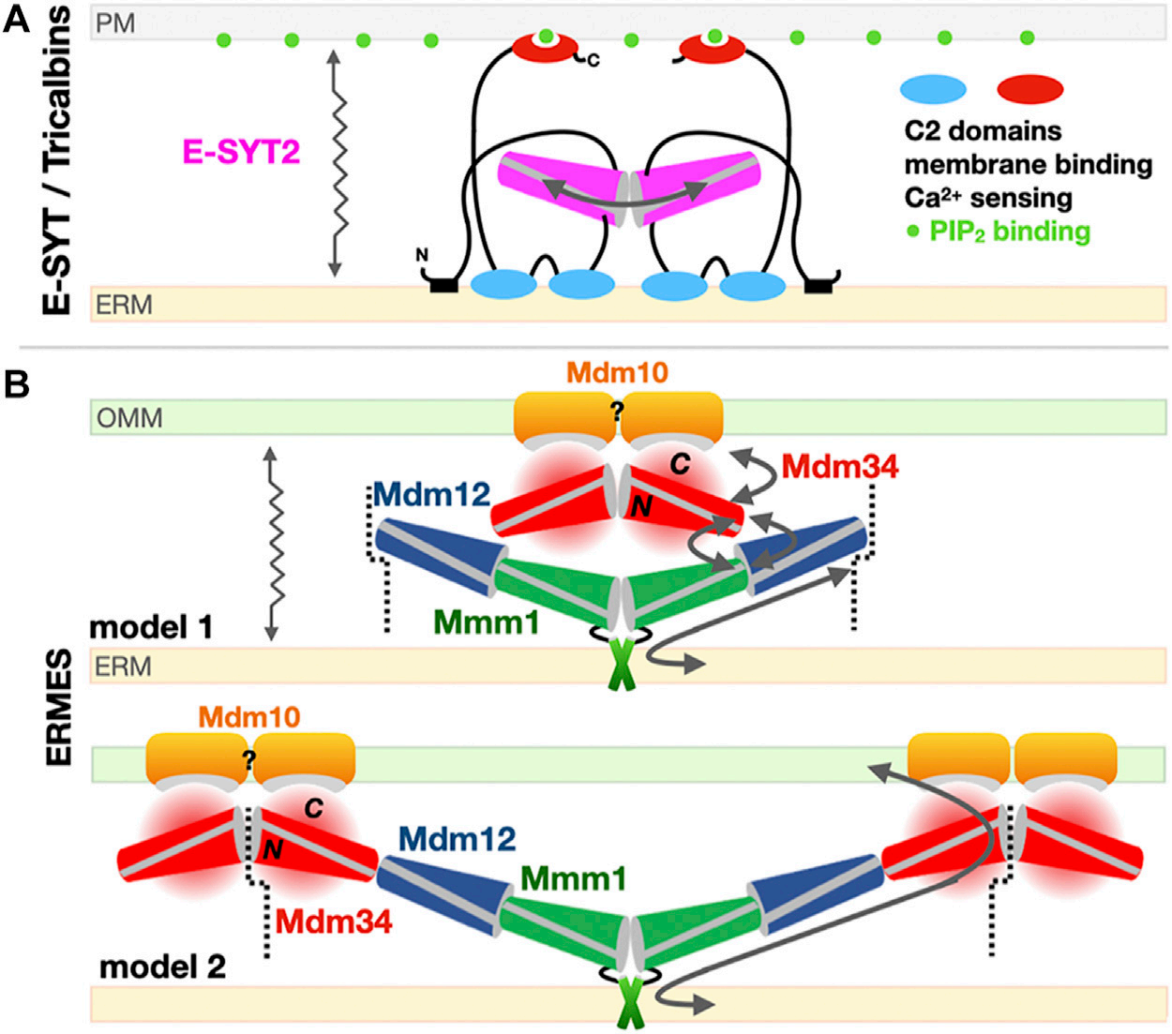
Models for the exchange of phospholipids by SMP domains at contact sites. **(A)** *Tethered shuttle* model for SMP-mediated PL exchange by E-SYT2 (yeast Tricalbins) at ER-PM contact sites. E-SYT2 forms dimers *via* its central SMP domain and is tethered to both membranes (ER and PM) *via* N-terminal hydrophobic segments and three C-terminal C2 membrane binding domains. The most C-terminal C2 domain binds to PIP_2_ (PM) and is regulated by cytosolic Ca^2+^ levels. Given its dimensions a sole dimer of E-SYT SMPs *cannot* directly bridge one membrane to the other; the flexible linkers are long enough to enable the back-and-forth movement of the dimerized SMPs between the two membranes, alternatively extracting and releasing PLs. **(B)** Possible “*shuttle with handover*” versus “*lipid conduit*”models of assembly of the three SMP domains in ERMES at ER-Mitochondrion contact sites. Based on the Mdm12/Mmm1 SMP tetramer structure and the reported dimeric association state of Mdm34 SMP, two models can be proposed. Mdm10 is shown as a possible dimer while Gem1 has been omitted for simplicity. The dotted line indicates a possible mode of pseudo “infinite” polymerization of whole independent ERMES units supporting the formation of foci. For Mdm34, N and C refer to its N-terminal SMP domain and the uncharacterized and possibly disordered C-terminal region, respectively. The zigzagging arrows indicate that tethered-diffusion of SMP domains (in E-SYT2 and possibly in ERMES) is necessary to lipid transfer by enabling back-and-forth movement of their LTP domains between membranes.

E-SYT2 was the first SMP tether extensively characterized in structural, biophysical and cellular detail by the Reinisch and De Camilli groups (Giordano et al., 2013; Schauder et al., 2014; Fernández-Busnadiego et al., 2015; Saheki et al., 2016). A symmetrical dimer of E-SYT2 is tethered to the ER and PM through its two C2 calcium-binding domain (ER) and a PIP_2_ membrane-attachment domains (targeting it to the PM) while the two dimerized SMP domains shuttle glycerophospholipids between ER to PM along their concentration gradients. Based on these biophysical and structural data, it is thought that E-SYTs transfer lipids using an extraction-sliding-diffusion-release model where linker lengths and protein dimensions provide the necessary conformational freedom but preclude a transfer by direct bridging of the two organelles by the dimer of tubular SMP domains (Figure 7A).

For ERMES several models have been proposed, influenced by the tubular shape of the SMP domain and the presence of a long continuous hydrophobic channel spanning some 190 Å in the Mdm12-Mmm1 tetramer (Figure 7B) seemingly suited and sufficient for bridging the two apposed membranes and transferring lipids from one membrane to the other. These models however (Figure 7B) do not take into account the role of Mdm34, the last SMP-containing protein. Advanced homology modeling tools such as *Phyre* ^*2*^ (Kelley et al., 2015) and more recently *AlphaFold* (Callaway, 2020; Jumper et al., 2021) (https://alphafold.ebi.ac.uk/entry/P53083) validate the presence of the SMP fold in Mdm34. *In vitro* biochemical studies (AhYoung et al., 2015) suggest a weak/transient interaction between a dimer of Mdm34 SMP domains and the Mdm12/Mmm1 stable tetramer, hindering attempts to reconstitute a ternary ERMES-SMP complex in absence of Mdm10. This also suggests that SMP domains not only function as LTPs but also as versatile scaffolds to establish specific protein-protein interfaces. A more holistic model including Mdm34 (model 2 in Figure 7B) suggests the existence of a longer tubular assembly, forming a quasi-continuous tunnel connecting both organelles; this model accounts for the two-fold symmetry within the Mdm12-Mmm1 tetramer and also provides a mechanism explaining the congregation of ERMES molecules into foci. The toe-to-toe interaction we suggest between Mdm12 and Mdm34 would be weaker thus imparting the necessary flexibility and transience to the ternary assembly. An alternative model combining “lipid-sliding” in Mdm12/Mmm1 and a “hand-over” step to Mdm34 is also possible (model 1 in Figure 7B). If correct, “quasi-conduit” mechanisms like this seem well-suited to support the transfer “*en masse*” of lipids necessary to supply dynamic membrane structures such as mitochondria. This is better exemplified by the tether/bridge-LTP systems Vps13 and Atg2 described in Section 5 of this review. Although well-documented, the mechanism for the formation of foci where ERMES complexes congregate is not understood; Rasul et al. recently described the effects of a small single transmembrane spanning from the outer mitochondrial membrane in the yeast *Schizosaccharomyces pombe*, ERMES regulator 1 (Emr1), in regulating the number of ERMES foci (Rasul et al., 2021).

### 4.2 Mdm10: A Multi-Functional Mitochondrial Porin

**FIGURE 8:**
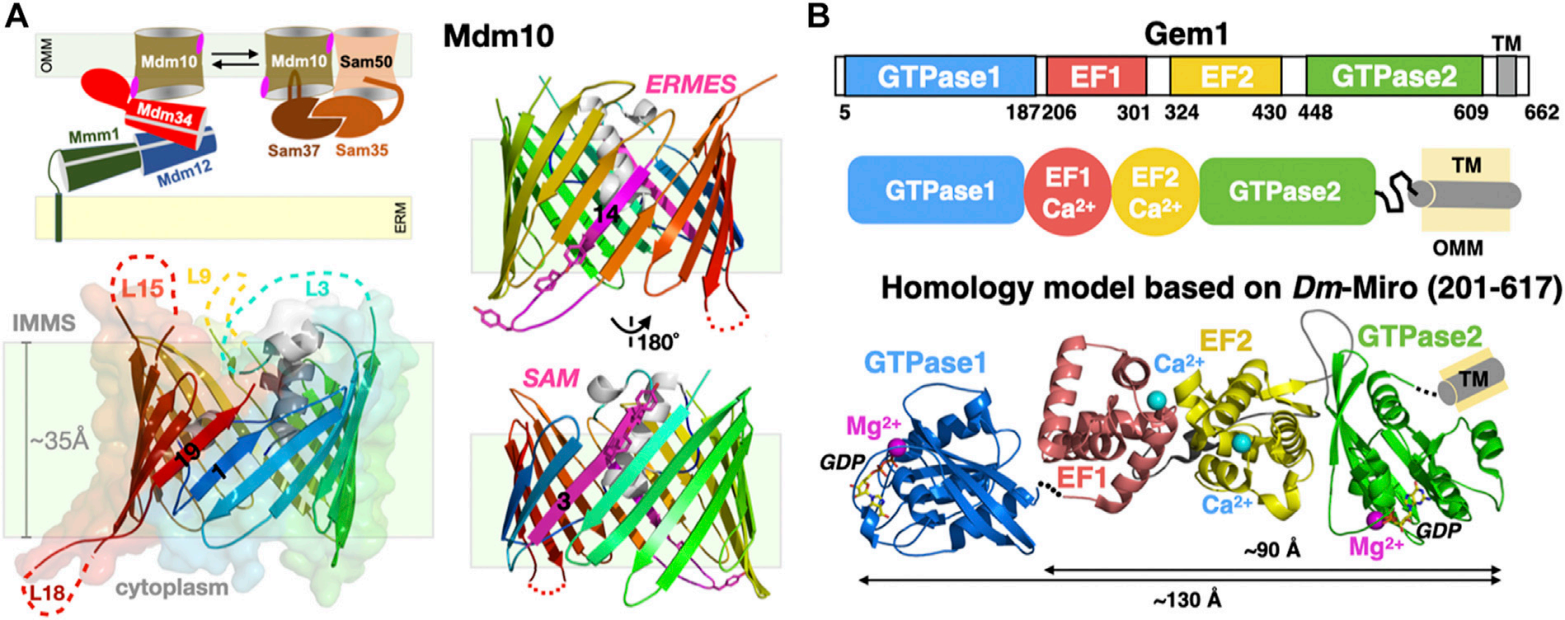
Mitochondrial subunits of ERMES: Porin Mdm10 and Rho1 GTPase Gem1. **(A)** Mdm10 serves dual functions as component of ERMES and SAM. Transmembrane 19-stranded β barrel structure of Mdm10. The 19 β-strands are colored using a rainbow pattern and strands 1 and 19 forming the lateral gate of the barrel are labeled. Unresolved loops in the cryo-EM structure are indicated; L18 is the only large cytoplasmic loop present in Mdm10. Two views of the Mdm10 barrel colored in rainbow pattern except for the two opposite binding interfaces for ERMES (Y296-F298 and Y301) and SAM (Y73 and Y75) colored in magenta. **(B)** Domain organization of the tail-anchored mitochondrial GTPase Gem1 and *Phyre* ^*2*^ composite homology model based on the *Drosophila/Homo* Miro partial X-ray structures. Domains EF1, EF2 and C-GTPase2 domains are modelled “*en blo”* based on the *Drosophila/Homo* Miro structures while the N-GTPase1 domain is modelled based on the *Homo* Miro structure, although its exact relative orientation within the full-length Miro structure is not precisely known. Ca^2+^ (magenta) and Mg^2+^ (cyan) ions are drawn as spheres (PDB 7BTY-6D71-6KTY).

The exact roles and mechanisms of action of Mdm10 within the ERMES remain unknown. However, genetic studies point at a connection between mitochondria-associated membranes and mitochondrial protein biogenesis (as in import and insertion) (Ellenrieder et al., 2017; Meisinger et al., 2007). In yeast, the porin Mdm10 is also a subunit of the Sorting Assembly Machinery (SAM) complex involved in the biogenesis and insertion of many outer mitochondrial porins (such as VDAC). The cryo-EM structure of yeast Mdm10 was recently revealed as a component of the SAM complex, in association with another mitochondrial porin, Sam50, and its two associated peripheral membrane proteins Sam35 and Sam37 (Takeda et al., 2021) (Figure 8A). Porin Mdm10 is composed of 19 β-strands arranged into a canonical transmembrane β-barrel (Flinner et al., 2013); with this last structure, the entire protein superfamily of mitochondrial porins has now been surveyed (Zeth, 2010). The three largest soluble loops of Mdm10, one cytoplasmic and two in the inter-mitochondrial compartment, were too flexible to be resolved in the cryo-EM structure (Figure 8A). Ellenrieder et al. characterized the molecular interactions existing between ERMES and SAM and showed that the two machineries bind to opposite faces of the porin barrel and concluded that Mdm10 acts as the mitochondrial integral membrane anchor for ERMES and its SMP-containing components (Ellenrieder et al., 2016). When mapped to the available cryo-EM structure of Mdm10, its ERMES-binding surface corresponds to β-strand 14 and the following short cytoplasmic loop L14 while the SAM-interacting surface corresponds to β-strand 3. Anecdotally though, Ellenrieder et al. used a poor homology model to erroneously map the two interaction areas on the same cytoplasmic side of the outer mitochondrial membrane (Ellenrieder et al., 2016); the now-available Mdm10 structure indicates that the ERMES-binding interface is on the cytoplasmic leaflet while its SAM-binding interface rather lays within the internal leaflet of the outer mitochondrial membrane (Figure 8A). Although deletions of various Mdm10 loops do not seem to affect cell viability or interactions with SAM and ERMES, it still remains unclear whether the largest cytoplasmic loop (L18) of Mdm10 might also engage in additional direct molecular interactions with the soluble SMP core of ERMES (i.e., Mmm1-Mdm12-Mdm34) and/or the C-terminal uncharacterized region of Mdm34, respectively. In the recent SAM structure, the Sam37 peripheral subunit interacts with one of the short *α*-helices sitting at the center of the transmembrane β-barrel of Mdm10. Although the studies from the Pfanner and Becker groups hint at close interactions between Mdm10 and Mdm34, the molecular details are still unknown (Ellenrieder et al., 2016). Lastly, it is not excluded that Mdm10 might form dimers or tetramers with membrane-bending or at least membrane-reshaping/destabilizing properties with possible effects on the kinetics of lipid desorption and exchange also discussed in Section 7 of this review; this has been observed for Tom40, another 19 β-strand porin and main subunit of the translocase of the outer membrane (TOM) complex (Tucker and Park, 2019). These observations further complicate attempts to define subunit stoichiometry within an ERMES functional and structural unit.

### 4.3 Gem1: A Regulatory Subunit?

The fifth ERMES subunit Gem1 is the orthologue of mitochondrial Rho1 GTPase (Miro) in Metazoans (Figure 8B). This regulatory calcium-binding GTPase is a tail-anchored membrane protein, i.e., harboring a single C-terminal transmembrane helix. It was identified in the genetic screen performed by Kornmann et al. (2009) and later also detected in proteomic analysis of native ERMES complexes affinity-purified using tagged Mmm1 subunits (Stroud et al., 2011). High resolution crystal structures of *Drosophila* and human Miro (Klosowiak et al., 2013; Klosowiak et al., 2016; Smith et al., 2020) provide excellent homology modeling templates for yeast Gem1, a modular protein containing two functional ras-like GTPase domains flanking two calcium-binding EF hands (Koshiba et al., 2011) (Figure 8B). Its function seems regulatory; *in vivo* studies show that the GTPase1 and the two EF domains of Gem1 are required for its association with ERMES while the GTPase2 is required for proper PL exchange (Frederick et al., 2004; Kornmann et al., 2011; Koshiba et al., 2011). More recently Gem1 and ERMES have been involved in the formation of a cellular structure, the mitochondrial-derived compartment, in response to amino acid over-abundance (English et al., 2020). In Metazoans, Miro is a substrate for the kinase Pink1 and the E3 ubiquitin ligase Parkin and is essential to Ca^2+^-dependent axonal transport of mitochondria along microtubules.

## 5 Lipid Transfer in Bulk by Protein Bridges and Tunnels

### 5.1 Proteins Vps13 and Atg2: Moving Lipids From ER to Expanding Double Membrane Structures

**FIGURE 9:**
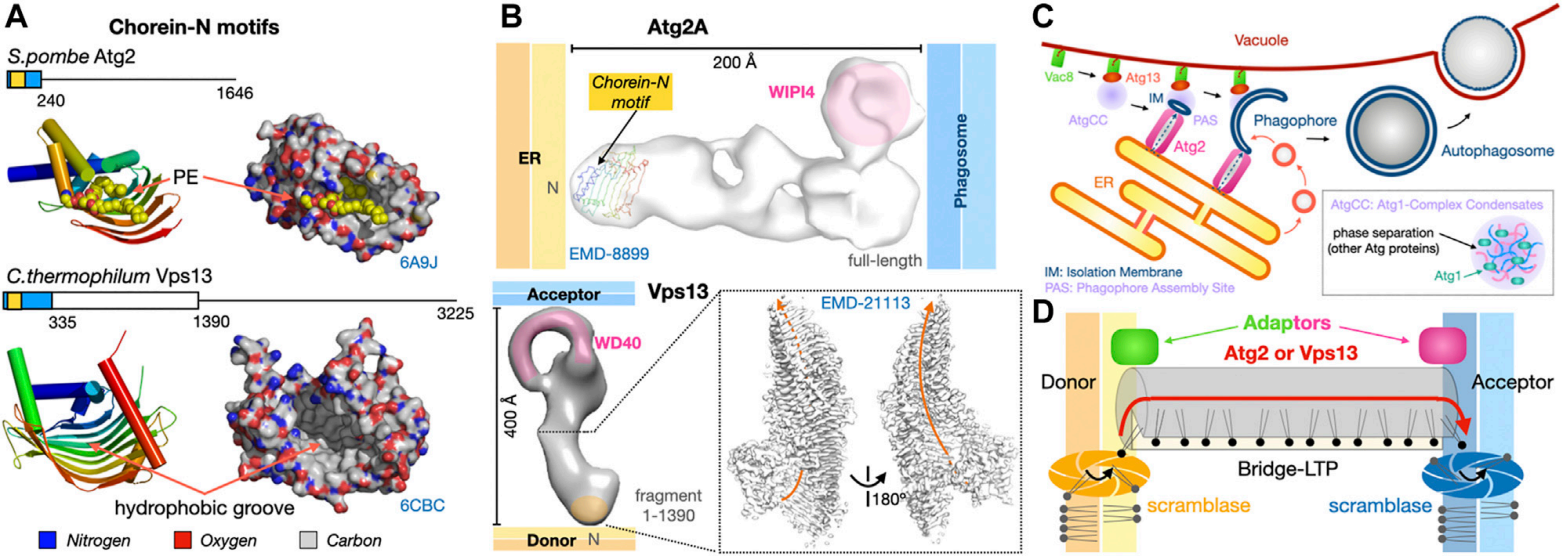
Dual tethers/LTPs Vps13 and Atg2 form bridges to transfer lipids in bulk at membrane contact sites. **(A)**. Schematic structure of the Atg2 and Vps13 proteins from *S. pombe* and yeast are shown together with the crystal structures of the N-terminal fragments of *S. pombe* Atg2 (21-246) (PDB 6A9J) and *C. thermophilum* Vps13 (1–335) (PDB 6CBC) containing the Chorein-N motifs (∼100 residues boxed in yellow). Areas boxed in blue correspond to crystallized fragments. **(B)** NS-EM density map (30 Å resolution EMD-8899) of full-length (1-1938) human Atg2A bound to WIPI4. NS-EM density map (30 Å resolution) of full-length (1-3144) yeast Vps13 and cryo-EM map (3.8 Å resolution EMD-21113) of the N-terminal fragment (1-1390) of Vps13 from C. *thermophilum*. The cryo-EM map of *Ct*-Vps13 reveals the presence of a central groove suited for the transfer of PLs (orange arrow). The relative position occupied by the crystallized fragment containing the Chorein-N motif is shown in hAtg2A. Membranes and proteins are drawn to scale. The WIPI adaptor protein bound to Atg2A or WD40-like domain of Vps13 are highlighted in pink. **(C)** Bulk autophagy in yeast localizes at the ER-vacuole interface. Vacuolar protein 8 (Vac8) and Atg13 localize the Atg1-Complex Condensates near the membrane where Atg1 activation by phosphorylation triggers the formation of an isolation membrane (IM) at the phagophore assembly site (PAS). The LTP-bridge Atg2 fuels phagophore expansion by channeling ER PLs in synergy with vesicular accretion. Matured autophagosomes subsequently fuse with the vacuole and their content is then degraded/recycled. **(D)** “Lipid sink” model for the bulk transfer of lipids by Atg2 and Vps13. For Atg2, scramblases in the donor and acceptor membranes drive exchange by re-equilibrating lipid concentrations between leaflets.

Some cellular processes such as mitochondrial biogenesis and autophagy can require production of large amounts of biological membranes on relatively large physical scales and short time scales. Autophagy is a complex and highly coordinated process (Hollenstein et al., 2019; Hollenstein and Kraft, 2020) involving the formation of a phagophore assembly site (PAS), a transient structure that expands into the autophagosome, a double membrane compartment that grows from lipids supplied by the ER to eventually engulf cytoplasmic macromolecules and organelles for their delivery to lysosomes for destruction/recycling. In yeast, protein Atg2 is involved in autophagy, transporting lipids from the ER to the expanding autophagosome (Valverde et al., 2019; Ktistakis, 2019; Osawa and Noda, 2019; Otomo and Maeda, 2019). In yeast bulk autophagy, the early PAS is formed by high-order assemblies of the Atg1 kinase complex with other Atg proteins to form a so-called Atg-complex condensate (Hawkins and Klionsky, 2020; Fujioka and Noda, 2021) (Figure 9C) where LLPS organizes the site of autophagosome formation (Fujioka et al., 2020). In the past years crystallography and cryo-EM have provided considerable insights into the molecular interactions between Atg proteins and membranes at the heart of PAS formation and autophagosome biogenesis (Lai et al., 2019). Still in yeast, protein Vps13 is found at vacuole-mitochondria (vCLAMP) and vacuole-ER (NVJ, the nuclear envelope derives from the ER) contact sites (Lang et al., 2015) and also promotes the formation of another double membrane structure, the prospore (Park and Neiman, 2012). Both proteins are evolutionarily conserved. In humans, they are two Atg2 homologues (Atg2A-B) while four Vps13 homologues (Vps13A-D) are thought to transfer lipids from ER to other organelles (Kumar et al., 2018) such as mitochondria, endo-lysosomes (Muñoz-Braceras et al., 2019) and LDs (Wang et al., 2021); membrane association and cellular localization are determined by a combination of structural motifs present within each protein (*e.g.*, the WD-40 like domain in yeast Vps13) and the interaction with membrane-specific adaptor proteins binding at the C-terminal and N-terminal ends of the protein (e.g., WIPI in Atg2A and Spo71/73 in yeast Vps13) (Kumar et al., 2018; Dziurdzik and Conibear, 2021; Nakamura et al., 2021) (Figure 9B).

Both proteins are large ranging from ∼1500 residues for Atg2 to ∼3000 residues for Vps13 in Fungi. A number of structural and biochemical studies have shed light on the mechanisms of lipid transfer and membrane/organelle tethering by Atg2 (Valverde et al., 2019; Maeda et al., 2019; Osawa et al., 2019) and Vps13 (Kumar et al., 2018). Several low resolution (∼30 Å) NS-EM reconstructions of yeast Vps13 (De et al., 2017), human Atg2A and yeast Atg2 (Chowdhury et al., 2018), a 15 Å resolution cryo-EM analysis of human Atg2A (Valverde et al., 2019) and a 3.5 Å resolution cryo-EM structure of the N-terminal half (1390 residue-long) of *Chaetomium thermophilum* Vps13 (Li et al., 2020) revealed that both proteins form elongated structures capable of tethering and bridging two membranes (Figure 9B). Sub-3 Å resolution crystal structures of N-terminal fragments of *Schizosaccharomyces pombe* Atg2 (Osawa et al., 2019) and *C. thermophilum* Vps13 (Kumar et al., 2018) reveal that the signature chorein-N motifs (∼100 residues) adopt a U-shaped structure with a hydrophobic groove that can accommodate PL acyl chains to mediate lipid transfer (Figure 9A). These motifs are located at the N-terminal tip of the elongated Atg2 and Vps13 EM reconstructions (Figure 9B). Remarkably, the near-atomic resolution cryo-EM reconstruction of the N-terminal half of fungal Vps13 published by the Reinisch group reveals the existence of a hydrophobic groove (Figure 9B), extending well beyond the mere Chorein-N domain and suited for the “sliding-channeling” of phospholipids in bulk.

Atg2 and Vps13 both function as tethers bridging heterotypic membranes and as LTPs using a “tunnel” mechanism to channel lipids between membranes (Leonzino et al., 2021; Lees and Reinisch, 2020; Reinisch and Prinz, 2021). Mechanistically, they bypass the protein diffusion step described in Sections 2 and 3. They appear quite promiscuous as they bind most glycerophospholipid types and enable the efficient bulk transfer of PLs to support the expansion of the acceptor membranes (in particular for the autophagosome). Mutations introduced within the hydrophobic tunnel block lipid movements but not tethering, thus supporting this bridge model where Atg2 and Vps13 transfer lipids in bulk from the ER towards the expanding autophagosome or between organelles, respectively (Osawa et al., 2019; Valverde et al., 2019; Li et al., 2020) (Figure 9D). Since bulk lipid transfer is essential to support processes involving membrane expansion (such as phagosome growth) but also to connect organelles such as mitochondria and plastids that are essentially disconnected from vesicular trafficking pathways, it is possible that ERMES might serve a similar function to meet the substantial lipid requirements of the mitochondrial network in yeast. The mechanisms determining directionality of these lipid transfer processes will be discussed in Section 7.

### 5.2 Protein Bridges or Conduits Transferring Lipids are not a Eukaryotic Specialty

**FIGURE 10:**
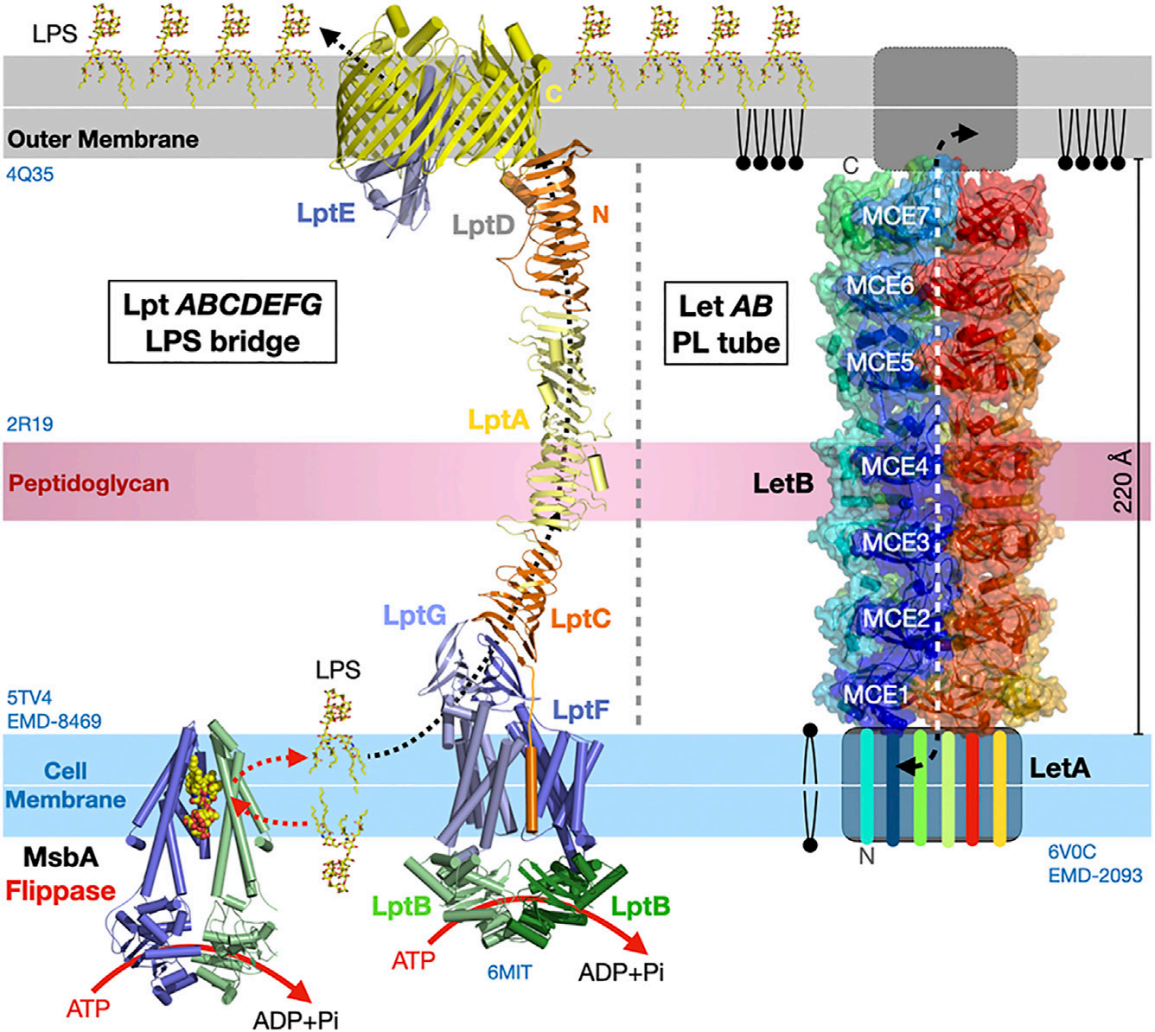
Bulk lipid transfer systems in Gram-negative bacteria. Structure of the Lpt bridge transferring LPS (PDB 5TV4 2R19 4Q35 6MIT EMD-8469) and the LetB proteinaceous nanotube transferring PLs between membranes in Gram-negative bacteria (PDB 6V0C EMD-20993). The β-jellyrolls domains present in Lpt C, F, G, A and D align to form a periplasmic bridge with a solvent-exposed V-shaped hydrophobic groove. Hexamers of LetB form a protein tubular structure with a central channel capable of binding PLs and spanning the periplasmic space. The six LetB chains are colored in a rainbow pattern. The seven MCE domains repeated along one chain (in blue) are labeled. The sides of the protein tube are porous enabling solvent to enter the channel. It is unclear what drives PL transfer in the Let system. Structures and membranes are drawn to scale.

Vps13 and Atg2 are the only two eukaryotic membrane-bridging systems extensively characterized so far. Bacterial organisms also seem to have evolved similar strategies to transport lipids “*en masse*” between two membrane compartments although the proteins assemblies and structures involved are not evolutionarily related. In Gram-negative bacteria, the lipopolysaccharide (LPS) transport system (Lpt) (Suits et al., 2008; Qiao et al., 2014; Botos et al., 2016; Owens et al., 2019) and the lipophilic envelope-spanning tunnel (Let) (Isom et al., 2020) span the entire periplasmic space to transfer LPS or PLs from the cell membrane towards the outer membrane and across the thin peptidoglycan layer, respectively. In Lpt the β-jellyroll folds present in LptA, the periplasmic C-terminal domains of LptC and LptF, and the periplasmic N-terminal domain of the outer transmembrane β-barrel translocon LptD interact and align to form a periplasmic bridge with a solvent exposed V-shaped hydrophobic groove suited for the transfer of LPS (Figure 10). Transport is powered by ATP hydrolysis (Mi et al., 2017; Owens et al., 2019) that ensures unidirectional vectorial transfer by flipping and pushing in the LPS substrates from the cell membrane into the lipid bridge. In Let where each LetB chain consists of seven repeats of a mammalian cell entry (MCE) domain, six chains of LetB associate to stack seven hexameric MCE rings on top of each other and create a protein tubular structure spanning the periplasmic space. Pore-lining loops in each MCE domains delineate a central channel capable of binding PLs (Figure 10).

## 6 Lipid Droplets and Seipins: Desorbing Lipids Between Bi- and Mono- Layers

Lipid droplets are organelles that store neutral lipids. Remarkably, they are delineated by a single monolayer of lipids instead of one (or several) bilayer(s) like all other eukaryotic organelles. The main function of LDs is to serve as storage compartment for fatty acids. Neutral lipids (i.e., triacylglycerides (TAGs) and sterol-esters (SEs)) stored in LDs are used for energy and membrane biogenesis when fatty acids are mobilized either by lipolysis or lipophagy of LDs in yeast (van Zutphen et al., 2014; Schepers and Behl, 2021). LDs are generated *de novo* from the ER through *protein-assisted desorption* of lipids to form a separate oil phase in the aqueous cytoplasm. Phase transition in microdomains of the ER membrane are essential to the nucleation and growth of LDs. LD-organelle contact sites have distinctives features that set them apart from all other MCSs where proteinaceous tethers appose membranes but prevent their fusion. This is not the case for MCSs with LDs, in particular at the biogenesis stage, where the two membranes (double layer for the organelle and hemi-layer for the LD do merge through hemi-fusion intermediates or lipidic bridges (Schuldiner and Bohnert, 2017).

**FIGURE 11:**
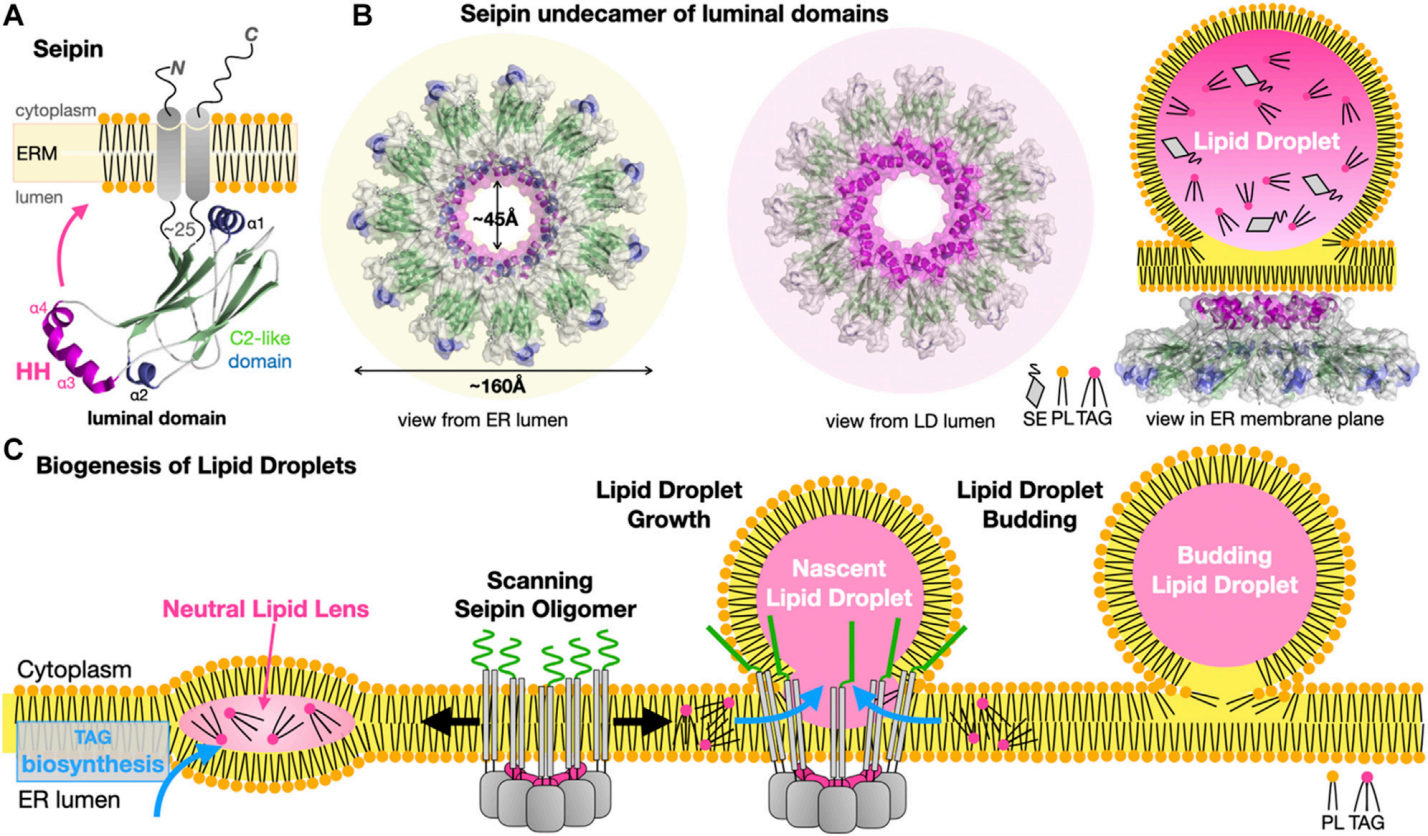
Seipins at the Endoplasmic Reticulum-Lipid Droplet interface. **(A)** Structure of the *Hs*/*Dm* seipin monomers. Two transmembrane helices anchor a luminal domain to the ER. The evolutionarily conserved β-sandwich domain (or C2-like) contains a membrane-anchoring hydrophobic helical (HH) insertion (magenta). **(B)** Undecamer of *Hs*-seipin (*Dm* forms similar dodecamers) shown in transparent surface representation highlighting the luminal HH α-helices involved in ER anchoring. Three views from the ER and LD lumens and, sideways in the ER membrane plane, are shown to emphasize the cup- or funnel- shaped structure and likely mode of membrane association of the seipin oligomers on the ER-luminal side of a nascent LD. Secondary structure elements are colored the same way in **(A, B)**. **(C)** Model for the biogenesis of lipid droplets from their nucleation to their growth. Oligomers of seipin (grey) scan the ER membrane lateral plane for the presence of neutral lipid lenses. The HH motifs (magenta) acts as a scaffold regulating the surface tension while the N-terminal extensions (green) further stabilize the seipin-nascent LD MCS. The luminal domain of seipin facilitates the lateral transfer of TAGs to the growing LD (PDB 6MLU-6DS5 EMD-9146 EMD-8909).

Seipins have been identified at the ER-LD interface in Metazoans. Dysfunctional seipin in humans is associated with type 2 Berardinelli-Seip congenital lipodystrophy, a severe disease characterized by the loss of adipose tissue, together with severe insulin resistance, leading to ectopic storage of fat elsewhere in the body (e.g., muscles and liver) with dramatic consequences on health. Thus, seipin regulates lipid storage at the cellular *and* organismal levels. Seipins regulate ER-LD contacts and growth by stabilizing ER-LD contacts and facilitating the incorporation of proteins (lipid biosynthetic enzymes) and lipids into the growing LDs (Salo et al., 2016). The cryo-EM structures of the seipin from *Drosophila melanogaster (Dm)* (Sui et al., 2018) and *Homo sapiens* (*Hs*) (Yan et al., 2018) were the first to provide valuable insights into the architecture of the ER-LD MCS and some mechanistic aspects of LD biogenesis. Seipins have two transmembrane helices flanking a highly conserved luminal domain consisting of an eight stranded β-sandwich (quasi-immunoglobulin-like) resembling the lipid-binding domain of Niemann-Pick type C2 protein that binds sterols (Xu et al., 2007); the domain contains an insertion of two hydrophobic helices (HH) that function as ER-attachment helices (Figure 11A). The cryo-EM structures reveal that 11 or 12 copies of seipin oligomerize to form a cup- or funnel- shaped ring structure in the human or in the fly, respectively (Figure 11B). The C2-like domain of *Hs*-seipin binds anionic PLs such as PA with a preference for unsaturated acyl chains (Yan et al., 2018) suggesting a role in the lateral transfer of TAGs to the nascent LD.

A general model has emerged explaining the role of seipins in LD biogenesis at ER-LD contacts (Figure 11C). Biosynthesis of TAGs in the ER membrane results in the accumulation and phase exclusion of TAGs into a neutral lipid “oil lens” between ER leaflets; accumulation of PL packing defects at the ER membrane surface and alteration of its surface tension and curvature are probably sensed by oligomers of seipin scanning the bilayer surface. Seipin oligomers localize at the lens further stabilizing and fueling the growth of the resulting nascent LD by facilitating incorporation of TAGs by lateral diffusion. More recently, the cryo-EM structure of the yeast Sei1/Ldb16 seipin complex provides remarkable insights into LD biogenesis (Klug et al., 2021). Sei1 forms homodecameric rings similar to those observed in metazoan seipin structures; however, in contrast with its metazoan homologues Sei1 cannot concentrate TAGs without its ER membrane partner Ldb16 that initiates the recruitment of TAGs later enhanced by transmembrane segments of Sei1 stabilized upon interaction with Ldb16. Following budding, LD growth and fusion with other LDs continue, supported by the recruitment of new membrane-interacting enzymes or adaptor proteins, respectively (Walther et al., 2017; Olzmann and Carvalho, 2019).

LDs do not constitute “dead ends”; following their formation at and separation from the ER membrane they establish many additional contacts with other organelles such as mitochondria, peroxisomes, lysosomes (vacuoles) and other LDs (Olzmann and Carvalho, 2019). In starving cells, fatty acid trafficking occurs through LD lipolysis, autophagy but also mitochondrial fusion dynamics pointing at the role of LD-mitochondrion/ER MCSs (Rambold et al., 2015). LDs are also connected to peroxisomes to relieve LDs from damage induced through lipid peroxidation (Chang et al., 2019). Some of the proteinaceous machineries and the associated molecular mechanisms involved in the formation, maintenance and dynamics of these other LD-organelle contact sites have been characterized (Freyre et al., 2019; Olzmann and Carvalho, 2019; Choudhary and Schneiter, 2021; Renne and Hariri, 2021).

## 7 Vectoring and Energizing Lipid Exchange at Membrane Contact Sites and Within Membranes: “Passive” *Versus* “Active” Exchanges and Counter-flows. Lipid Gradients and Membrane Asymmetry

What are the driving forces for the exchange and transport of lipids? The lipid transfer functions of many of the proteins described here have been dissected using *in vitro* systems with liposomes used as donor and acceptors of fluorescent lipids to monitor their LTP-mediated transfer either by dequenching or quenching of fluorescence; liposome flotation has also been used to characterize the affinity of LTP for membranes of specific compositions. Studies of the SMP domains of E-SYT2 (Saheki et al., 2016), Mdm12 and Mmm1 in ERMES (Jeong et al., 2017; Kawano et al., 2017; AhYoung and Egea, 2019), the Ups1/Mdm35 (Watanabe et al., 2015) and also Osh6 (Maeda et al., 2013; Moser von Filseck et al., 2015) and OSBP (Mesmin et al., 2013) are examples of these approaches. Other studies have used partially purified intact membrane fractions to monitor lipid transfer (Kojima et al., 2016) or genetic and mutagenesis methods combined to lipidomics to study the effect of lipid biosynthesis rewiring on the levels and distributions of cellular lipids in the network of organelles (John Peter et al., 2021; Peter et al., 2021).

### 7.1 Energetics and Directionality of Lipid Transfer by Lipid Transfer Proteins

**FIGURE 12:**
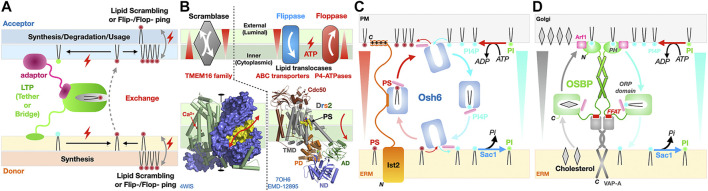
Directionality and energetics of lipid transfer and formation of gradients and membrane asymmetry. **(A)** General factors determining directionality of lipid transfer at MCS. A hypothetical LTP is depicted here as directly tethered to the donor membrane and attached to the acceptor membrane *via* an adaptor protein. **(B)** Scramblases are bi-directional and equilibrate lipid concentrations between leaflets while flippases/floppases require ATP hydrolysis to transport lipids unidirectionally against the gradient to generate membrane asymmetry. Symmetric dimer of the fungal Ca^2+^ activated-TMEM16 lipid scramblase from *Nectria haematococca* shown as a solvent accessible surface (blue) to emphasize the presence of a hydrophilic cleft (colored in yellow) running along the side of each monomer and spanning the entire width of the membrane; the other monomer is represented as green cylinders with its two calcium ions positioned the middle plane of the membrane (red spheres) that might assist neutralizing negative charges on the PL during its transfer (PDB 4WIS). Structure of yeast P4-ATPase PS flippase Drs2-Cdc50 chaperone complex with its transmembrane (TMD), nucleotide-binding (ND), phosphorylation (PD) and actuator (AD) domains of the flippase labeled. A molecule of PS bound mid-membrane along the putative translocation path is visible (PDB 7OH6 EMD-12895). **(C, D)** Lipid counterflow transport by tethered LTPs; **(C)** PS/PI4P counterflow by Osh6 at ER-to-PM contact sites where Ist2 [only represented on the left half of panel **(C)**] is the ER-to-PM tether that interacts with the soluble LTP Osh6 to localize its lipid transfer activity at this MCS. **(D)** Cholesterol/PI4P counterflow by dimers of OSBP at ER-to-Golgi contact sites. PI, phosphatidylinositol; PI4P, phosphatidylinositol 4-monophosphate. Directionality of lipid transfer is indicated by the cyclic colored arrows while gradients of PS, PI4P and cholesterol are indicated by shaded triangles. ATP-consuming biosynthesis provides PI4P while its irreversible hydrolysis into PI by the ER-associated phosphatase Sac1 sets the directionality of these two cycles.

The term transport should be used with caution. In integral membrane protein biology, transport implies an active process, requiring an energy source and therefore its expenditure, usually in the form of ATP (e.g., ABC transporters such as endosomal ABCG1 that transports sterols), or ion-gradients (e.g., proton gradient) generating membrane potentials. Such transporters are opposed to channels that “only” facilitate the movement of molecules along an existing concentration gradient (e.g., aquaporins water channels). Thus, transporters can function against concentration gradients while channels cannot (Li et al., 2021). In molecular terms, most LTPs are passive and do not directly expend energy (i.e., nucleotide hydrolysis or membrane potential gradient). Enzymes responsible for specific steps along the synthesis/transformation or catabolism of lipids are specifically distributed in specific organelles; thus, metabolic reactions taking place between compartments can drive LTP-mediated exchange of lipids along concentration gradients (Shiino et al., 2020) (Figure 12A). Ceramide transport by CERT from ER to Golgi is driven by the transformation of ceramide into sphingomyelin in the Golgi that cannot be transported back. LD growth is probably the most spectacular example showing the result of the activities of TAG synthesis in the ER and of LD-membrane attached or embedded enzymes on the generation and subsequent growth of these droplets, respectively (Figure 11).

Two classes of integral membrane proteins participate in the transfer of lipids from one leaflet to another within the same membrane: scramblases and flippases/floppases. Technically they are not considered as lipid “transfer” proteins as they do not shuttle lipids across an aqueous space between membranes. Their functional importance is considerable as they abolish (i.e., scramblases) or establish (i.e., flippases and floppases) membrane asymmetry and contribute to lipid fluxes (in particular PS) at MCSs and within the cell through the formation of gradients between leaflets within a membrane and thus support the formation of lateral nanodomains not only within the membrane but also within leaflets (Shin and Takatsu, 2020; Lenoir et al., 2021) (Figures 12A,B). Flippases transport lipids (sterols, glycerophospholipids such as PS, PE or PC and ceramides) from the exoplasmic (external or luminal) to the cytoplasmic leaflet while floppases work in the opposite direction; these processes require ATP hydrolysis to enable the vectorial transport against a concentration gradient. In yeast and Mammals, ABC transporters (Prasad et al., 2016; Kumari et al., 2021; Banerjee et al., 2021) and P4 subtype ATPases (P4-ATPases) (Shin and Takatsu, 2020) are largely represented among lipid translocators (flippases/floppases). ABC lipid translocases play a role in bacterial lipid transport systems such as the Lpt bridge that requires the MsbA LPS-flippase to energize the process in addition to the ATP hydrolysis activity of subunits LptB (Owens et al., 2019; Mi et al., 2017) (Figure 10). P4-ATPases constitute another important group of lipid flippases whose structures and mechanisms of action have been recently elucidated in the case of yeast PS flippase Drs2/Cdc50 (Bai et al., 2019; Timcenko et al., 2019; Timcenko et al., 2021) and human homologues ATP8A-Cdc50 (Hiraizumi et al., 2019) and ATP11C-Cdc50 (Nakanishi et al., 2020a; Nakanishi et al., 2020b).

Scramblases equilibrate lipid distributions between leaflets and thus dissipate lipid asymmetry in membranes; they are ATP-independent and follow energy potential gradients associated with a simple chemical concentration gradient or the membrane electric potential (resulting from charge distributions) (Lenoir et al., 2021). The first scramblase structure belongs to the TMEM16 protein family (Brunner et al., 2014; Brunner et al., 2016) and reveals a dimer where each monomer bears a membrane-spanning hydrophilic cleft facing the lipid environment. Scramblases most likely combine membrane thinning and electrostatic shielding mechanisms to lower the energetic barrier associated with transferring a PL molecule, and in particular its polar headgroup, from one leaflet to the other, by providing a trans-bilayer hydrophilic path for its facilitated diffusion down the concentration gradient (Brunner et al., 2014; Brunner et al., 2016; Theorin et al., 2019) (Figure 12B). Recently, a model proposed that lipid scramblases present in the donor (i.e., ER scramblases VMP1 and TMEM41B) and acceptor (i.e., Golgi scramblase Atg9A) membranes tethered by the mammalian Atg2A tether/LTP support autophagosome expansion by re-equilibrating lipid distributions between leaflets of donor and acceptor membranes and compensate for their respective depletion or enrichment (Figure 9D) (Ghanbarpour et al., 2021). Scramblase Atg9 is the only Atg integral membrane protein (Guardia et al., 2020; Maeda et al., 2020; Matoba et al., 2020); Atg9-vesicles derived from the Golgi are essential as they provide the initial membranous kernel of the isolation membrane (IM or phagophore) (Mari et al., 2010; Yamamoto et al., 2012; Matoba and Noda, 2020; Orii et al., 2021), a cup-shaped membrane structure that further expands into the autophagosome using lipids supplied from the ER by Atg2 (Figure 9C). In this “molecular sink” model, lipids are pushed out at the donor site and pulled in at the acceptor site as a result of synergistic scramblase activities in both organelles.

Compared to scramblases that facilitate lipid movements at rates of ∼10,000 lipids/sec, flippases are much slower (∼2–25 lipids/sec) (Goren et al., 2014); this is due to fundamentally different molecular mechanisms: flippases of the ABC-transporter class use an alternating access mechanism involving large conformational movements and domain rearrangements powered by the energy associated with ATP binding and hydrolysis that has been extensively studied and characterized in transporters belonging to the ABC and the Major Facilitator Superfamily classes (Li et al., 2021). P4-ATPases also undergo ATP-driven large conformational rearrangements (Hiraizumi et al., 2019; Nakanishi et al., 2020a; Timcenko et al., 2021) (Figure 12B).

Osh6 (Moser von Filseck et al., 2015), Osh4 (von Filseck et al., 2015) and OSBP are two examples illustrating how tethered LTPs can simultaneously support the counterflow of two distinct lipids at a membrane contact site using chemical gradient energy. The LTP Osh6 consists solely of an ORP domain (Figure 3) while OSBP contains an ORP domain but also a PH domain and FFAT motif involved in dual membrane tethering through direct interaction with PI4P in the PM and adaptor-mediated attachment to the ER membrane, respectively (Figures 12C,D). Both proteins are capable to bind two different lipids (PS/PI4P and Cholesterol/PI4P, respectively) and transfer them in opposite directions. While Ohs6 functions as an LTP tethered at ER-PM junctions by the Ist2 protein (D'Ambrosio et al., 2020) (Figure 12B), OSBP attaches itself to the Golgi through its PI4P-binding PH domain and also tethers to the ER through its FFAT motif interacting with the ER-anchored vesicle-associated membrane protein-associated protein A (VAP-A) (Figure 12D). Their elegant mechanisms have been dissected in exquisite detail by the Drin (Moser von Filseck et al., 2015) and Antonny (Mesmin et al., 2013; Antonny et al., 2018) groups, respectively. ATP-consuming biosynthesis generates PI4P for its exchange from PM/Golgi to ER while its *irreversible* hydrolysis into PI by the ER-associated phosphatase Sac1 sets the directionality of these two cycles. OSBP has a higher affinity towards PI4P than the sterol so exchange of sterol for PI4P is facilitated at the Golgi; once the PI4P is shuttled to the ER, its hydrolysis acts as an energy sink, irreversibly driving this branch of the cycle. This counter-exchange mechanism is remarkable as it enables transport of cholesterol from the ER to enrich the Golgi. These latter examples underline the importance and complementarity of LTPs next to vesicular trafficking as LTPs by transferring lipids against their concentration gradients can counteract lipid homogenization resulting from vesicular traffic (Antonny et al., 2018).

### 7.2 Other Factors Determining the Fluxes of Lipid Exchange

In lipid exchange mediated by “basket”-shaped LTPs that shuttle lipids back and forth by diffusion or tethered diffusion, the rate-limiting step for the transfer of a lipid from one membrane to another is the extraction or desorption from the membrane itself rather than the LTP-assisted transport (Dittman and Menon, 2017; Wong et al., 2017). Spontaneous desorption of a lipid into the aqueous phase and henceforth between two membranes is energetically costly (∼10–20 kCal/mol) and extremely slow (several days) (Rogers et al., 2021). Local membrane composition, lipid packing and membrane order also affects the energetics of lipid desorption (Rogers et al., 2021). LTPs lower the desorption energy barrier as they partition the lipid from a bilayer into a hydrophobic binding pocket instead of bulk aqueous solvent. Electrostatics and desolvation/solvation of the lipid-binding site (Lipp et al., 2019) and the membrane reshaping or bending properties of LTPs are thus crucial for desorption at the donor membrane. The curvatures observed in the E-SYT2 dimer and Mdm12-Mmm1 tetramer, might modulate membrane shape (and/or *vice-versa*) thus lowering the activation energy associated with lipid desorption and transfer to the LTP core.

**FIGURE 13:**
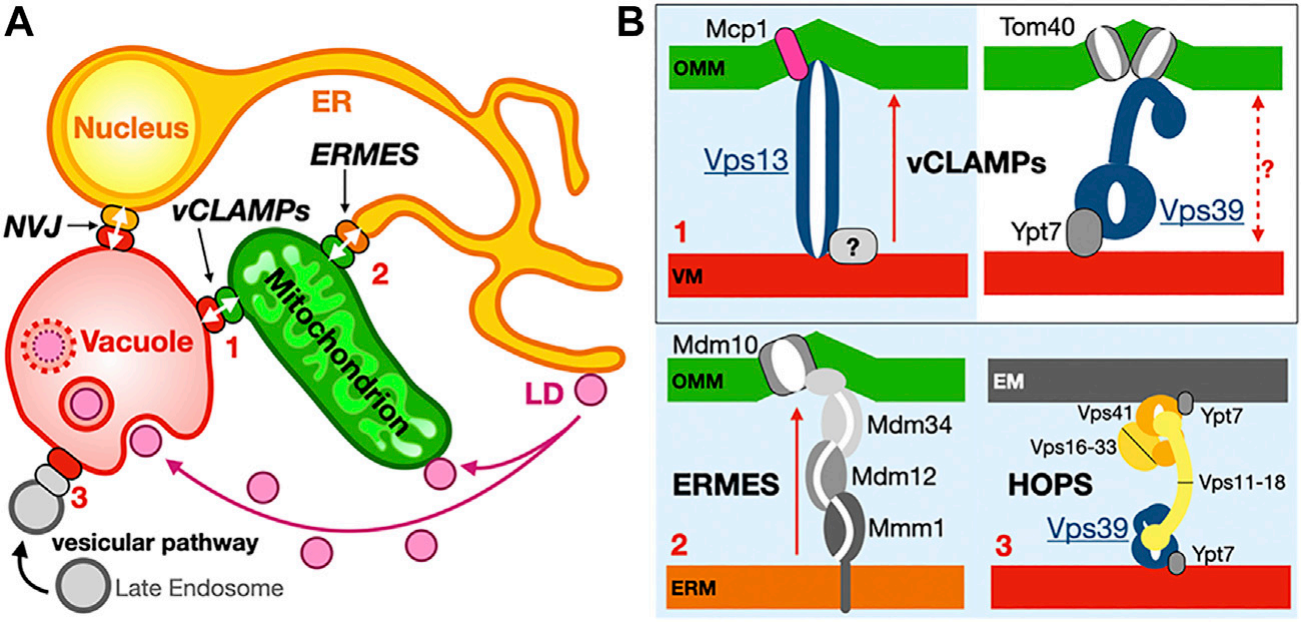
Redundancy of membrane contact sites. **(A)** Overall schematic recapitulating the interconnectivity of organelles in the case of the ER (perinuclear ER included), mitochondria and vacuole implicating ERMES, vCLAMP and NVJ contacts sites. LD are connected to mitochondria and vacuole (shown here *via* lipophagy) while the vesicular network also connects to the vacuole through a Vps39-HOPS complex redundant to the Vps13-vCLAMP and ERMES. **(B)** Two distinct vCLAMPs use different subsets of proteins, Vps13 or Vps39, and respectively include proteins Mcp1 or porin Tom40 that could affect lipid desorption through membrane distortion although lipid exchange has not been associated with *Vps39-vCLAMP*. The ERMES, Vps13-vCLAMP and Vps39-HOPS pathways are redundant. Vps13 is also found at the NVJ under certain conditions. Complexes boxed in light blue constitute redundant pathways. Red arrows indicate lipid exchange activity.

*Redundant yet slow and insufficient? When a lot is not enough.* Based mostly on *in vitro* reconstitution studies and measurements, at the single protein molecule level these systems appear quite slow (∼1 lipid/sec at best) and likely involved in local adjustment/tweaking of the lipid composition of membranes. For example, *in vitro*, Osh4 transports sterol or PI4P (in absence of their counterpart) at rates ∼ 2 lipids/min; however, when both two lipids are present, Osh4 counter-exchanges them at rates of ∼ 20 lipids/min (von Filseck et al., 2015). CERT transport ceramides at ∼ 4 lipids/min (Hanada et al., 2003). Even when relative abundance and localization of these LTPs are factored in order to calculate the global lipid exchange rate at a specific MCS, the estimated fluxes seem despairingly too slow to support cellular functions. Thus, other factors (either unknown or not easily measurable/quantifiable in the experimental conditions used *in vitro* or *in vivo*) have to be considered. In the case of ERMES and lipid exchange at yeast mitochondria-associated membranes, Petrungaro and Kornmann attempt to address these discrepancies (∼500 fold difference) to reconcile Biology with the apparent insufficiency of measured lipid fluxes (Petrungaro and Kornmann, 2019). They propose that the presence of distinct LTPs combined with the existence of bypasses/shunts, well-exemplified by the vCLAMP and ERMES case discussed in Section 4.1, could account for these discrepancies (Figure 13A). They also suggest that membrane “destabilizing” proteins such as Mcp1 (John Peter et al., 2017) could further facilitate lipid desorption; as discussed in Section 4.2, the Mdm10 ERMES porin could also function as a catalyst of lipid desorption by locally distorting/thinning the membrane. Interestingly, another porin the outer mitochondrial protein translocase Tom40 is the receptor for Vps39 in one of the two functionally independent complexes identified at vCLAMPs (González Montoro et al., 2018) (Figure 13B). Tom40 barrels associate into dimers and even tetramers and induce membrane bending (Bausewein et al., 2017; Araiso et al., 2019; Wang et al., 2020). The importance of membrane distortion and thinning induced by transmembrane proteins (either α-helix or β-strand based) has been documented in different systems, in particular protein translocation systems (Li et al., 2021; Wu and Rapoport, 2021). The same argument could be made with LTP-associated scramblases that manipulate the local biophysical properties of membranes to move lipids between leaflets.

What about transfer by lipid bridges/tunnels then? Theoretically they bypass or at least minimize protein diffusion effects leaving us to essentially consider *lipid dynamics within the proteinaceous tunnel* and *lipid desorption* as sole apparent limiting factors. Molecular dynamic simulations should prove insightful, although they would require better-defined atomic models of these eukaryotic protein bridges and model their interaction with membrane surfaces. As it stands now, lipid exchange systems such as Atg2 and Vps13, and possibly the more contentious ERMES, would need to sustain much faster rates of transfer, in order to support biological processes requiring fast and/or massive membrane expansion during organelle biogenesis such as phagosome growth and mitochondrial fusion or fission. These considerations beg one question: How complete is our knowledge of all the LTP systems and lipid exchange routes and networks operating in a given organism even seemingly as “simple” and genetically tractable as yeast?

## 8 Perspectives and Conclusion: Membrane-Bound or Membrane-Less but Never Contact-Less

**FIGURE 14:**
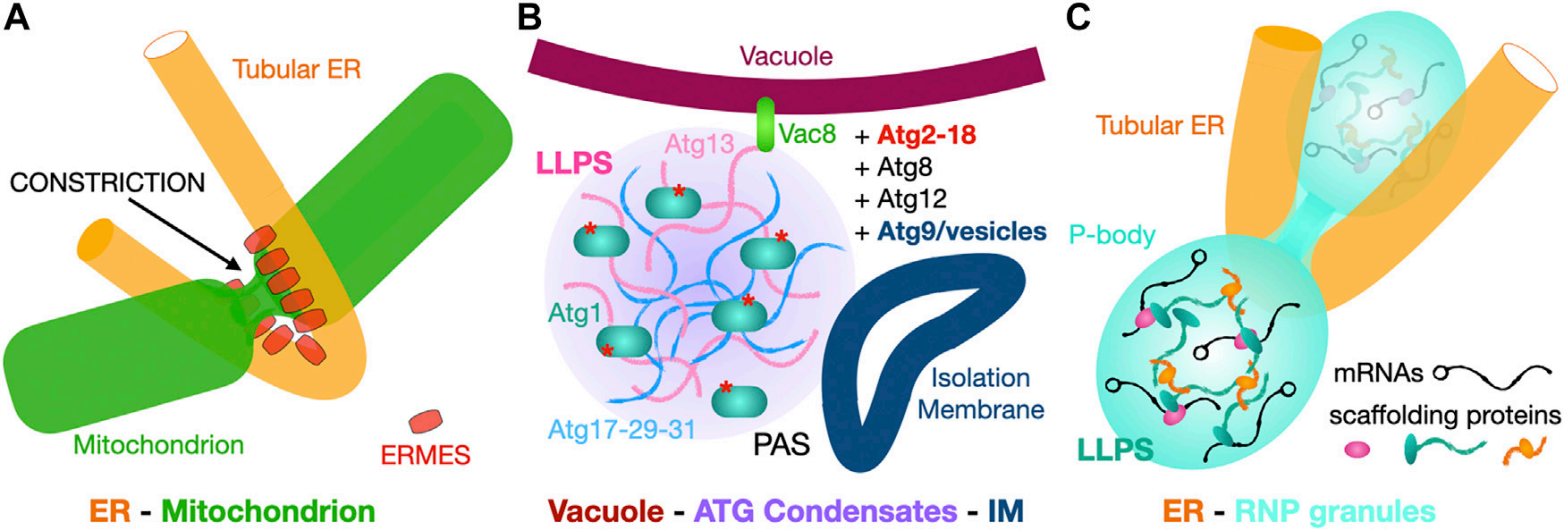
Membrane and membrane-less contact sites. **(A)** “Classic” membrane to membrane contact site. The ER tubular network contacts mitochondria at constrictions marking their division. In yeast tethers such as ERMES are involved at these sites. **(B, C)**. Membrane to membrane-less contact sites. **(B)** Vacuole-to-Atg complex condensate interactions drive isolation membrane formation at the ER-Vacuole-PAS contact site. Scramblase Atg9-vesicles derived from the Golgi provide the initial IM (phagophore) that will later expand into the autophagosome using ER lipids supplied by Atg2. **(C)** The ER tubular network contacts membrane-less P-bodies and regulates their biogenesis and activity. Simplified schematic of the content of PBs with RNPs made of repressed mRNA associated with multivalent scaffolding proteins that drive LLPS and formation of this membrane-less compartment.

The ER acts as a nexus for many cellular processes including membrane protein and lipid biogenesis and establishes direct contacts with all other organelles; the Lippincott-Schwartz group recently described differential phase behavior of ER membranes depending on the organelle contact sites (King et al., 2020). While this review focuses mostly on the role of yeast and metazoan MCSs in lipid transfer, it is reductionist and simplistic in the case of ER-to-Mitochondrion contact sites. The ER is not only the main site for lipid biosynthesis, contacting virtually all other organelles in the cell, it is also the main storage compartment for cellular Ca^2+^ a key signal in cellular physiology (Marchi et al., 2017). Mitochondrial division occurs at positions where tubular ER contacts mitochondria and constriction occurs prior to the recruitment of the division/fission machinery (Friedman et al., 2011; Murley et al., 2013; English et al., 2020) (Figure 14A). Thus, tether-mediated interactions between the ER and other “classical” membrane-bound organelles not only mediate the efficient transfer of metabolites and ions but also serve as hubs to control organelle biogenesis and regulate cell death (Giorgi et al., 2018; Marchi et al., 2018). MCSs are studied in the model eukaryote organism *S. cerevisiae* but also in human cells and these studies have uncovered the links between dysfunctional MCSs and diseases such as neurological diseases (e.g., Parkinson and Alzheimer), “metabolic” diseases (e.g., diabetes/obesity) and cancer (Henne, 2017; Reinisch and Prinz, 2021).

We mentioned the role of intrinsically disordered regions (IDRs) in proteins in the formation of membrane-less organelles in the introduction; this theme in Biology has received more attention in the recent years. IDRs in soluble or membrane-bound proteins might also play a role in controlling protein segregation, dynamics and orientation in or near crowded membrane environments. A N-terminal 90-residue long IDR in OSBP controls the orientation and dynamics of OSBP at ER-to-Golgi MCSs (Jamecna et al., 2019) and prevents its homotypic (Golgi-to-Golgi) abortive tethering. In a broader biological context, similar mechanisms might be at work to segregate lipid transporters from nutrient channels and vacuolar protein secretion complexes (Egea, 2020; Garten et al., 2020) in the narrow parasitophorous vacuole of the malaria pathogen *Plasmodium* and other related apicomplexan parasites, at the interface between the parasitic plasma membrane and the vacuole surrounding the parasite within the infected red blood cell. Low-complexity IDRs might act as entropic barriers limiting protein density, restraining protein orientation and thus facilitating protein mobility in the narrow and crowded environment of MCSs.

Biomolecular condensates have emerged as organizers of cellular biochemistry; these liquid-like compartments reversibly self-assemble through weak and multivalent interactions as multivalent proteins containing LCRs and or IDRs are prone to such behavior (Li et al., 2012; Banani et al., 2017; Lyon et al., 2021). As mentioned in Section 5 LLPS organizes the site of autophagosome formation (Fujioka et al., 2020). Localized molecular interactions trigger nucleation of the PAS at the interface between vacuole and ER (Hollenstein et al., 2019) where the Atg machinery sequentially assembles to provide the isolation membrane kernel that further expands into the autophagosome in yeast bulk autophagy (Figure 14B); similar features indicative of the role of MCSs in the regulation of autophagy also apply to mammalian cells (Kohler et al., 2020). Other such condensates include processing-bodies (PBs) and stress granules, membrane-less “organelles” that undergo mysterious fission processes. PBs contain RNPs made of non-translating (repressed) mRNAs associated with proteins controlling their processing and decay. Multivalent weak intermolecular interactions and the presence of IDRs in these “scaffolding” proteins that mediate protein-protein *and* protein-RNA association drive PB formation through LLPS. The proteinaceous machinery that controls PB assembly and partitioning from the cytoplasm is conserved between yeast and humans (Luo et al., 2018). In human cells, the Voelz group has recently observed and analyzed the direct interaction between the tubular ER network and PBs and shown that the ER controls the proliferation and the fission of these membrane-less compartments (Kornmann and Weis, 2020; Lee et al., 2020) (Figure 14C), reminiscent of what is observed at mitochondrial fission contact sites (Figure 14A).

These new discoveries pave the way for exciting research to unravel the molecular mechanisms controlling these complex interactions and their integration at the cellular level. Thanks to the technical developments in EM (cryo-EM and cryo-ET) and other advanced cellular imaging methods improving resolution and sensitivity in the last decade, more complex MCSs with their associated tethers, LTPs and scaffolding regulatory factors will become tractable targets for structural and cell biologists, promising further insights into the architecture of the eukaryotic organelle network and a better understanding of diseases linked to dysfunctional MCSs.

## Glossary

ABC: ATP-binding cassette
Atg: autophagy-related
Atg2: autophagy-related protein 2
BPI: bactericidal/permeability inducing protein
BSCL2: *Berardinelli-Seip* congenital lipodystrophy 2
Cryo-EM: cryo-electron microscopy
Cryo-ET: cryo-electron tomography
CETP: cholesteryl-ester transfer protein
ER: endoplasmic reticulum
ERMES: endoplasmic reticulum-mitochondrion encounter structure
E-SYT: extended synaptotagmin
FFAT: two phenylalanines in an acidic tract
HOPS: homotypic fusion and protein sorting
IDR: intrinsically disordered protein region
IM: isolation membrane
IMMS: inter-mitochondrial membrane space
Lam: lipid transfer protein anchored a membrane contact site
LCR: low complexity region
LD: lipid droplet
Let: lipid envelope spanning tunnel
LLPS: liquid-liquid phase separation
LPS: lipopolysaccharide
Lpt: lipopolysaccharide transfer
LTP: lipid transfer protein
MCE: mammalian cell entry
MCS: membrane contact site
NS-EM: negative stain electron microscopy
NVJ: nucleus-to-vacuole junction
ORP: oxysterol-binding related protein
OSBP: oxysterol-binding protein
Osh: oxysterol-binding homology
P4-ATPase: P4 subtype P-ATPase
PAS: phagophore assembly site
PDZD8: PDZ domain-containing protein 8
PH: pleckstrin homology
PI4P: phosphatidyl inositol 4-monophosphate
PIP_2_: phosphatidyl inositol (4, 5)-diphosphate
PM: plasma membrane
PL: phospholipid
SAM: sorting assembly machinery
SE: sterol ester
SMP: synaptotagmin-like mitochondrial lipid-binding protein
StarKin: StART-like proteins
StART: steroidogenic acute regulatory protein-related lipid transfer
TAG: triacylglyceride
TULIP: tubular lipid-binding protein
VAP: vesicle-associated membrane protein-associated protein
vCLAMP: vacuole and mitochondria patch
Vps13: vacuolar protein sorting-associated protein 13
WIPI: WD-repeat protein interacting with phosphoinositides
